# Troubling the Canon: Language, Geography, and the Politics of STS Publishing in Five Decades of Journal Publications

**DOI:** 10.1177/03063127251386080

**Published:** 2025-12-12

**Authors:** Maurizio Meloni, Ayuba Issaka, Sam Cadman, Benjamin Hegarty, Luca Chiapperino, Tessa Moll

**Affiliations:** 1 School of Humanities and Social Sciences, Deakin University, Burwood, VIC, Australia; 2 Global Centre for Preventive Health and Nutrition (GLOBE), Institute for Health Transformation, Deakin University, Burwood, VIC, Australia; 3 School of Social Sciences, Monash University, Clayton, VIC, Australia; 4 The Kirby Institute, UNSW, Sydney, Australia; 5 STS Lab, Institute of Social Sciences, Faculty of Social and Political Sciences, University of Lausanne, Switzerland; 6 Department of Psychology, Faculty of Arts and Social Sciences, Stellenbosch University, Stellenbosch, South Africa

**Keywords:** STS canon, internalized symmetry, citational justice, epistemic pluralism, decolonizing

## Abstract

STS is now a global endeavour, with journals, scholars, and associations in a wide range of regions, languages, and transnational networks of knowledge. Building on recent debates about decolonizing research, we contribute to debates about epistemic injustice and asymmetries in the discipline by analysing a large sample of articles in 17 STS journals, published between 1976 and 2023. Our goal is to understand the multiplicity of factors that shape processes that lead to hegemony and canonization. Drawing on a database of 12,045 articles, we describe the language of publication and geography of authorship, in addition to the language policy of the journals and location of their editorial offices. We then analyse the 350 most cited articles (ca. 3%) in our sample and focus our analysis on the 40 most cited publications, looking at the processes of canon-formation in the discipline. We argue that while STS has always emphasized the situatedness of knowledge and promoted epistemic pluralism, there is still significant work to be done to analyse how asymmetries in publishing and circulation of knowledge take place. To address epistemic injustice, more needs to be done to undo the processes through which canon formation takes place, moving the field further away from the Euro-American networks of knowledge and power in which it is still primarily embedded.

Fifty or so years have passed since the emergence of Science and Technology Studies (STS) as a discrete—albeit blurred—field, with consolidated norms, debates, and citational practices. Seen from the vantage point of established Euro-American genealogies, the making of a ‘field of its own’ (Jasanoff, 2017) was established not merely as an intellectual project (Sismondo, 2010). STS also emerged in response to the needs of rapid post-World War II scientific development as a part of the Cold War-era military industrial complex. It was in this context that the first programs in ‘science, technology, and society’ were created in key United States universities (Cornell, Massachusetts Institute of Technology, University of Pennsylvania, Harvard, Stanford), later followed by European (since the 1970s), Australian (since the 1980s), and Asian and Latin American (since the 1990s) institutions (Fu, 2020). Since the early 2000s, STS has grown into a lively global and plural endeavour. The 4S 2022 meeting in Mexico (as with the one in Buenos Aires 2014) was conducted in three languages (Spanish, Portuguese, and English), and STS journals are publishing articles in different languages in Asia and Latin America. The Transnational STS Network serves as an important vehicle for reflecting and documenting the plural character of the field, connecting scholars and communities around the world writing in different languages (Fortun, 2018; Kaşdoğan & Fortun, 2020). One important strength of STS—now inscribed in journal aims, publishing norms, and citation practices—is an engagement with a wide array of regional and national traditions and support for a plurality of formats and languages. An editorial published in *Science, Technology, & Human Values* reflects a growing commitment to an STS that is inclusive of diverse voices (Neale et al., 2023). Speaking to ongoing decolonizing and feminist critiques, the editors remind potential contributors to consider to what extent their bibliography reflects STS as a diverse and transnational field. A journal like *Catalyst*, which regrettably is not included in our sample (see Limitations section), has not only featured special issues on science from the South (Amrute & Murillo, 2020) but in 2025 published ‘the first bilingual—en el sentido pocho—Special Section’ (English/Spanish) to appear in the journal, on the topic ‘Knowing borders: Materialidades, ecologías, estéticas’. The editors speak clearly of a desire to generate ‘a linguistic third space’ as ‘an intentional, insistent collective praxis’ (L. A. Smith et al., 2025, p. 5 and p. 12). Recent debates on STS in countries as different as Turkey (Alkan et al., 2023) and China (Santos et al., 2023), both with a longer tradition of institutionalized non-Western science and technology, or whole subcontinental regions ‘going South’ such as Latin America (Kreimer, 2022a, 2022b; Rodriguez Medina, 2018a, 2018b), India, South Africa, and East Asia (Anderson, 2012; Dumoulin Kevran et al., 2018; Fischer, 2016; Foster, 2016; NatureCulture, 2022), evidence this vitality as well as tensions with the Euro-American roots of the discipline.

Undoubtedly, this is a story of success and inclusion, but one marked by heavy contradictions and structural constraints. A rich body of scholarship has addressed these asymmetries and unequal development in the discipline at many levels (Fu, 2007; Kreimer, 2022a, 2022b; Rodriguez Medina, 2018b, 2024; Sariola, 2018), and analysed how the infrastructures of knowledge production (which include STS) are embedded within colonial and postcolonial histories (Anderson, 2002; Anderson & Adams, 2007; Harding, 1998; Turnbull, 2000; Verran, 2001). Other critical voices have highlighted the effects of the standardization of STS publishing norms (Kreimer, 2022a, 2022b; Law & Lin, 2017; Lin & Law, 2022; Lyons et al., 2017; Mikami, 2021), or the continued dominance of academics and publishers in the Global North (Invernizzi et al., 2022; Kreimer, 2022a, 2022b; Mikami, 2021; Rodriguez Medina, 2024), as well as the implications of Anglophone hegemony for epistemic production (Lin & Law, 2022; Lyons et al., 2017; Shineha et al., 2010). Two recent articles have stood out for their significance to our own project: Kaltenbrunner et al. (2022) on emerging publishing norms in STS; and Invernizzi et al. (2022) analysing a set of leading STS journals in the last decade (2010-2019), with a particular focus on Latin America, to demonstrate the ongoing hegemony of Euro-North American centres and the different fate of STS publications between centres and peripheries.

## Our contribution: Language, canon, and symmetry

In this article, we contribute to this unfolding debate about the multiplicity of factors in building patterns of hegemony in STS. We take a sample of STS journals (n = 17), in (roughly) the first half century of the discipline, 1976-2023, with 1976 selected as starting date among many competing options.^1^ We carry out this analysis in progressive steps, first looking at 12,045 articles in 17 journals, using bibliometric methods, in their different languages of publications (English, German, Spanish, Portuguese, Italian, French, Danish) and then focusing on the most cited articles within this sample, for which a qualitative analysis will be offered. Before going into the empirical details of our analysis and methodological choices, however, we explain our theoretical entry points to address, in a multifactorial way, how hegemony is constructed: through a) language, b) canon formation and its geography, and c) asymmetries and epistemic justice.

### Language

In his analysis of the rise of Global English as the *lingua franca* of science after WWII, Gordin (2015, p. 1) has described scientists as ‘the most resolutely monoglot international community the world has ever seen’. Given the peculiar relationship between STS and the world of science and technology, and also the fact that STS began to formalize in the mid-1970s, a time when Global English was on the rise, our first theoretical concern in the analysis was with linguistic pluralism in STS publications. The existence of a *lingua franca* has many advantages of course, but the problems generated by monolingualism are also very significant and known to all the members of the STS community who had to transition (in a conference, a paper, a book, or a whole career) from non-English to English: Things are not quite where they were supposed to be in the original language. Style is different, pressuring the speaker or writer to mimic something that is alien. Metaphors and analogies are culturally bound, linked with close experiences of personal upbringing, and often non-transferrable. Finally, what counts as a decisive argument is not clear, and in general cognitive norms of acceptance and strategy of persuasions can only be guessed and perhaps discerned after long years of experience (see Ramírez-Castañeda, 2020).

As with the embedded structural burdens of working in academia in English for the non-native speaker, there is the problem of cross-linguistic and cross-cultural definitions of key terms that reflects not only differences in meanings but differences in the relative power and prestige of languages that come to shape which meanings are taken up and operationalized in STS. As Appiah (cited in Hountondji, 2002, p. xix) notes, the meaning of ‘la science’ in French appeals to a ‘wider range of systematic knowledge than the natural or social sciences’. Japanese has linguistic concepts that facilitate different understandings of the relationship between the ‘social’ and ‘natural’ sciences than is assumed in much Anglophone literature (e.g. Imanishi et al., 2002). In his work on hunting in Zimbabwe, Mavhunga (2017) points to the definitional hegemony from the North on what counts as ‘science’ and ‘technology’ and therefore as the objects of STS interest (see also Neely & Meek, 2024). In this highly asymmetric framework, native or vernacular languages appear at best ‘consigned to a glossary’ (Mavhunga, 2017, p. 46) that can be used in a comparativist fashion to enrich Global STS.

On the other hand, working in a non-English language does not automatically achieve epistemic justice or escape problems posed by the effects of colonization. The Japanese case is a particularly useful reminder of how non-English national languages can inherit or be deeply embedded within colonial frameworks. Kondo and Swanson (2020), for example, consider how 鮭鱒論 (*sake masu ron*)—a Japanese concept that can be translated as ‘salmon trout theory’—while potentially useful to global multispecies and more-than-human theory, is nevertheless embedded within the historical dispossession of the Indigenous Ainu of northern Japan by the Japanese state. Different languages carry their own colonial and imperial histories, different from those of American and British histories of empire, or other European languages such as French, Spanish, or Portuguese that have also been key vehicles of modern colonial domination.

Thus, even the emancipatory potential of postcolonial/decolonial concepts in STS brings potential risks. As Pérez-Bustos notes of the Latin American context, the critical concepts that STS scholars use are often themselves paradigms that emerged through English-language debates in elite academic settings, in turn taking form within male-dominated scholarship in the region (Lyons et al., 2017, p. 41). For this reason, we selected our sample of 17 journals with the aim of testing how much these journals (editors, editorial boards, authors) had done historically to address these issues, pluralize language, make transparent the limitation and situatedness of knowledge, and avoid the trick of producing concepts and theories that seem to come from nowhere, concealing their debt to specific places and institutions.

### Canon formation and its geography

**Figure 1.**
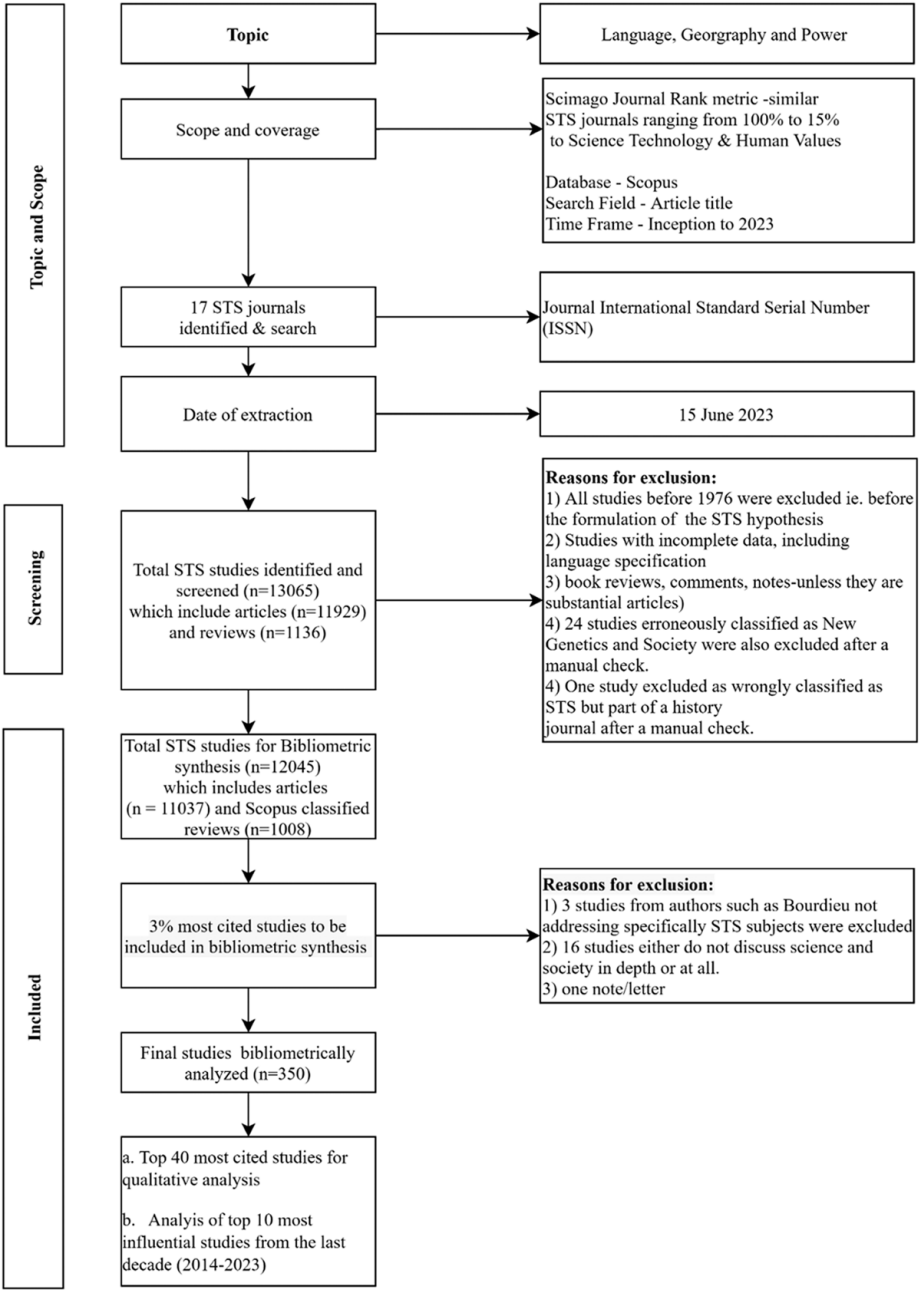
Flowchart search strategy.

As the reader will see, our empirical findings on language, while interesting, get us only to a limited level of granularity in our analysis. When we move to the second step of the research, the 3% most cited articles (see Figure 1), only one output is partially non-English (a trilingual article in French, English and Spanish). Questions about language were thus not granular enough to read critically through our sample. Our main theoretical concern has been to attend to *processes of canon-formation*, a topic that has become increasingly relevant in debates on decolonization in several disciplines (Alatas, 2022; Al-Hardan, 2018; Baber, 2003; Connell, 1997, 2019; Ehrmann, 2022) but which seems less prominent in STS so far. Interestingly, the term ‘canon’ is rare in our journal sample (c.f. Klein & Toledo Ferrera, 2024, for sociology). In addition, recent analysis of standardization and hegemony in STS (Invernizzi et al., 2022; Kaltenbrunner et al., 2022) has not focused explicitly on the most successful, cited, and hence somehow ‘canonical’ articles in the field.^2^ Beyond language, looking at canon-formation is an important exercise in self-reflexivity for any discipline: It enables us to investigate the importance of places, institutions, authors, tropes, and lemmas, that have gained and solidified extensive consensus and hence set the standards of the discipline in our 50-year sample. While possibly handbooks would give a more direct access to what, as an end-product, has become canonical in a field (see Issar, 2025), moving across 50 years of journal’s publications gives us an entrance into the dynamic moments through which certain paths and trends have prevailed over others and established future tracks for the field.

### Canon: A polysemic term with multiple filiations

The term ‘canon’ is an interesting one for STS scholars. From the Greek κάννα, a rod employed by craftsmen to measure things (similarly in semitic languages, for instance the Hebrew qāneh), the noun κανών, όνος comes; from it the masculine Latin cănon, ŏnis (origin of the English canon) assumes a number of meanings all of which are relevant to our present discussion . A cănon in Latin is: a) a marking tool or a measuring line and hence a model from which examples can be made; b) a tax to be paid annually to the emperor; c) and finally, since Christianity, a catalogue of sacred writings as established by the norms of a certain group (Grüning & Santoro, 2021; Lewis & Short, 1907). What starts as a humble measuring device turns then into something exemplar, next into a tax to pay in recognition of imperial or state powers (such as the so called *canone*, which Italians still pay these days to the public television system), and finally a body of writings endowed with celestial authority. This is nothing new for STS scholars; many in the field have in fact documented how categorization, standardization, and objectification order human interaction, generate subjects and subjectification, valorize some to the detriment of others, and include while excluding and making invisible, sometimes with epistemic violence.^3^ Undoubtedly, referring to an STS canon needs to be done carefully. A relatively young and epistemically hybrid discipline with blurred boundaries, STS has not developed canon processes comparable to more established fields like literature (Bloom, 1994), classics (Kennedy, 2013), or philosophy (Park, 2013). Nonetheless, what we record in our analysis is a significant process of narrowing down the multiplicity of languages (seven) and places (dozens) that we find in the wider sample of 12,045 articles. When we move from here to the most cited articles what we find is a *stricture* toward a narrow cluster of countries, institutions, and authors that count as first in the class and hence on the way to become canonized. This is, after all, another meaning of the Greek κάννα (kánna) and Latin canon: a reed, but also tube or pipe, from which the Latin canal or channel (*cannālis*/*canālis*) derives, as it does the Spanish *cañón* and the later English *canyon*. A canyon can be also called a *gorge* (also in French) or in Italian a *gola*, both literally meaning ‘throat’ with the sense of a stricture, a narrowing of the width, but also more broadly a steeper passage created by processes of erosion where water can flow more quickly because of the morphology of the site. As our data will show, if there is not a properly formalized canon in STS, there are, however, certainly canyons, gorges, and *gole*: structural processes of restriction and constraints that make some material stream faster than others. Another possible metaphor, not from geography but from evolutionary biology, would be that of the *bottleneck effect*, a drastic reduction in a population’s size and genetic diversity, often leading to increased vulnerability. A canyon of course can be seen as a geographic bottleneck that restricts traffic flow. Both metaphors are valid for our findings, albeit bottleneck effects often imply a randomness and speed in the bottlenecking event (natural disasters, etc) that is not relevant here.

### Symmetry and epistemic justice

Symmetry and epistemic justice are interconnected concepts that pertain to knowledge production, distribution, and validation. Being highly cited often reflects something about the value of an individual article, its impact on a debate, etc. However, when we look at a class of the most cited, and we see how narrow their geography, language, and academic affiliation are compared to the wider sample of all articles published in a certain field, it is fairly critical to ask if the system is not structurally biased to always favour the same networks of knowledge production. While we can recognize excellence and firsts in the class in every discipline, we should keep asking critically whether this occurs in parallel to the invisibilization of other excellences, styles of investigation, and norms of rationality.

In this perspective, Law and Lin have published several significant articles on the importance of expanding the principle of symmetry to a ‘postcolonial version of it’ (Law & Lin, 2017, p. 214). Extending their engagement with the inherent ‘Westernness’ of STS, we respond to their call for *a generalized symmetry* within the discipline itself that captures quite nicely the spirit of our attempt. Symmetry is a central and cherished principle in the history of SSK and STS (Bloor, 1976). One of its original goals was to undermine teleological explanations in the history of science and make possible more pluralistic ways of understanding ‘winners’ and ‘losers’ in knowledge production.

In what follows, these ideas of symmetry/asymmetry offer the rationale to probe the epistemic injustices in (a set of data representing) STS publications. The call towards internalizing symmetry in STS means for us to critically examine the proverbial economies that circulate within STS itself—the familiar aphorisms, canonical publications and case studies, as well as the citational habits that, as Singh and Lynch (2025) show, partake in the consolidation of the discipline’s identity, exclusions and hierarchies of recognition. In the discussion we briefly elaborate on the broader implications of internalizing symmetry in STS scholarship through citational justice and institutional inclusion. Recently, symmetry has been caught in debates on post-truth and erosion of trust toward experts (Fuller, 2016; Sismondo 2017). In our view, mobilizing the principle of symmetry in relation to epistemic justice instead (Lynch, 2017), and in dialogue with notions of asymmetries that have so deeply shaped dependence theory (Galassi, 2019; Kreimer 2022a, 2022b; Rodriguez Medina 2024), better captures its full democratic and pluriversalist political potential.

## Materials and methods

### Study design

To obtain a better perspective on linguistic and geographical dominance and processes of canon-formation in STS, we started by undertaking a bibliometric analysis of outputs in the field using the Scopus database and the SJR, *Scimago Journal and Country Rank* (simplified as Scimago below) journal ranking engine (Ali & Bano, 2021; Vaccaro et al., 2022). Scimago employs data obtained exclusively from Scopus and includes a specific ‘Find similar journals’ feature for each indexed journal.^4^ Given the blurred boundaries of STS, in using the ‘Find similar journals’ feature, we pragmatically designated the journal *Science, Technology, & Human Values* (ST&HV) as the base, given its official flagship role in 4S and hence integrative role within the field (see van den Besselaar, 2000). Utilizing the Scimago percentage of similarity metrics, which reflects Scimago’s methodology (Guerrero-Bote & Moya-Anegón, 2012), we selected journals that closely align with ST&HV, ranging from 100% to 15% similarity. On a search carried out on 15 June 2023, this process led to the identification of 17 journals within this similarity range: *Big Data and Society; BioSocieties; East Asian Science, Technology and Society; Journal of Science Communication; Life Sciences, Society and Policy; Minerva; Nanoethics; New Genetics and Society; Public Understanding of Science; Revue d’Anthropologie des Connaissances; Science and Technology Studies; Science as Culture; Science, Technology, & Human Values; Social Science Information; Social Studies of Science; Sociologias; Tapuya*. These journals formed the basis for data collection, analysis, and subsequent findings. The choice to privilege Scimago is not without problems, of course, particularly when it comes to mapping specific regional and national dynamics in knowledge production (Beigel, 2014; Beigel & Gallardo, 2021; Collyer, 2018) or in excluding important open access journals published on alternative platforms that are not indexed within Scopus (discussed further in the Limitations section). Moreover, Scimago has been broadly criticized for problems such as lack of transparency, variable coverage across time and different subject-matters, retrospective rankings modifications, opaque methodology, and mistaken country attributions (Mañana-Rodríguez, 2015; Mason & Singh, 2022). These are important issues and are part of a wider conversation on improving and pluralizing the process of assessment of research outputs (see, e.g., the San Francisco Declaration on Research Assessment, DORA, 2012, 2021). Recognizing the contingency and limitations of our final picture, we can also offer several arguments for the solidity of our findings. First, the Scopus records on which Scimago is based generally reflect the widest coverage of scientific literature worldwide (Mañana-Rodríguez, 2015; O’Neil, 2018). Particularly when it comes to canon-making in the contemporary world of research, where the prestige of books and book chapters is growingly replaced by indexed journal articles, including in the social sciences (Beigel, 2014; Kaltenbrunner et al., 2022), the Scopus records on which Scimago is based offers an excellent glimpse into the leading outputs in a certain field. Second, because of the blurred nature of STS and what contribution can be affiliated to the discipline, Scimago’s ‘Find similar journals’ feature offers a means of designating journals to the STS field that is as practicable, objectively determined, and reflective of prevailing institutional consensus as any presently available. Third, the sample of 17 journals we have obtained *is not particularly selective* or biased when it comes to language.^5^ It reflects publications in several different languages (in our sample: Portuguese, German, Italian, Spanish, French, Danish, and English) displaying a range of linguistic variation, though of course all these languages use the Latin alphabet and reflect the violent expansion of different European empires and colonial systems. Moreover, if we look at what are generally perceived to be major STS journals excluded because they are not indexed in Scopus, they all publish in English (*Catalyst—*with the exception noted above*—*and *Engaging Science, Technology and Society*). Three significant journals excluded from our search (because they are not captured within the 15% similarity threshold) are published outside of the Anglosphere, in Italy (*Tecnoscienza*), Mexico, and Spain, but only *Quipu* (last publication year 2000) and *Redes* fully publish in languages other than English (Spanish and Portuguese), while *Tecnoscienza* has increasingly replaced Italian with English since the first few years of its publication.^6^

### Search strategy

The search was conducted on 15 June 2023. Our search query used the International Standard Serial Numbers (ISSN) for the journals, providing a reliable method for identifying articles relevant to STS within each of the 17 journals (Verma et al., 2021). The search was focused exclusively on articles, without any language restrictions. Results were exported to a Microsoft Excel spreadsheet for subsequent analysis. Please refer to Figure 1 for an illustration of our search strategy.

### Criteria

First, we excluded studies conducted before 1976 (the year when the first issue of ST&HV, albeit in the form of a newsletter, was published). Second, we excluded studies that appeared in the search with substantially incomplete data, including language specification, such that it would not be possible to clearly identify them for the general sample. Third, we had to leave out the 1.7% of references that should be linked to Scopus records but do not in fact appear, and the further 0.1% that, as also highlighted in other studies, are not linked correctly (Baas et al., 2020).^7^ Fourth, we excluded notes (unless they appeared as a substantial article with footnotes etc), letters, and book reviews. Here we had one important manual control, as Scopus misclassified as ‘reviews’ hundreds of articles that are in fact full articles, or simply the first article of each issue.^8^ These multiple criteria resulted in a final dataset of 12,045 studies. A second exclusion process was applied in the analysis of the 350 most-cited studies. Here we excluded studies that, although published within these 17 journals, are not authored by scholars who can be defined as part of the STS community or are embedded within other disciplinary traditions.^9^ We are aware that these boundaries are not hard and fast, but this criterion helped to ensure that in the analyses of the top cited articles we focused only on articles that can be seen either as a) originating within debates in STS or b) contributing to topics that have relevance for STS debates.

### Limitations

In this bibliometric analysis, the Scopus database served as the primary source for collecting published articles. Scopus is widely recognized as one of the largest and most comprehensive databases of academic journal articles, providing essential publication data fields required for this study, such as article titles, affiliations, research areas, and author citations (Faruk et al., 2021). However, this approach to selecting journals has several limitations. Since Scimago exclusively draws on data from the Scopus database—a commercial abstract and citation database owned by Elsevier—it excludes journals that are not indexed for various reasons, including journal size, the use of commercial publishers (such as Wiley or Elsevier), and the language of publication. This also encompasses journals omitted due to factors as their size, peer-review process, or lack of affiliation with a commercial publisher. Unfortunately, this includes key journals such as *Catalyst* and *Engaging Science, Technology and Society*, and *Tecnoscienza.*

Another limitation is upstream. By following our sample of 17 journals, we have not only missed books and book chapters (for which another article would be necessary), but perhaps more significantly we do not capture a large number of key STS articles, by key STS authors, published in non-STS journals, or journals not captured by this search. For example, the highly influential ‘Some elements of a sociology of translation’ (Callon, 1984) appeared in English in *The Sociological Review* (not included in our corpus because it falls below the 15% threshold in the proximity search) and in French (1986) in *L'Année sociologique*. Similarly, the widely cited ‘Why has critique run out of steam’ (Latour, 2004) was published in *Critical Inquiry*. A number of other examples could be provided.^10^

Finally, given that algorithmic determination of similarity depends in Scimago on a combination of referencing vectors for each journal in Scimago’s dataset with vectors using a Jaccard-based algorithm,^11^ its results change over time. Our search, however, has remained stable for a significant period, (at least) from 12 June 2021 to 7 July 2024 making the 17 journals selected representative of the STS field during the planning of our project, when our data search was conducted on 15 June 2023, and following.

### Data analysis

The analysis consisted of four different steps. First, we reviewed the 17 journals’ histories, the geographic affiliations of the leading or chief editors (but not editorial board or office), and their multilingual outputs (publications of full articles in several languages, abstracts in several languages) or lack thereof. Second, we analysed all articles appearing in the Scopus search in these 17 journals (minus those excluded as per exclusion criteria above) with particular attention to language of publication, and authors’ geographical affiliations by country for the overall sample of 12,045.

This particular stage of our search, however, presents several limitations. Fewer than 90% of the articles have data available both for language or comparison across time and different decades. We had to manually adjust some language data resulting from a misrecognition of classification and languages by the database (a dozen of the French articles were read as German, something that is already telling of the biased nature of these infrastructures of knowledge when it comes to non-English languages). We also had to manually remove 24 studies attributed to *New Genetics and Society* (July 2024) that instead were in a biotechnology journal (*Genetic Engineer and Biotechnologist*). More substantially, many articles published in our sample of 17 journals were not obviously ‘STS’ articles: *Sociologias*, for instance, publishes classical sociological articles that do not discuss science and technology, or infrastructures of research and knowledge production. Similarly, a significant number of papers in *Public Understanding of Science* also refer more to media studies than STS as such. Given the limitation of the search and the number of manual interventions, this step of the search could be used only for a very general overview of broad trends in STS. We gathered from these results an overall sense of geographic and linguistic complexity in the discipline over 50 years, and a picture of the language policy and geography of editorial offices of the 17 journals, but nothing more. To answer more granular questions about canon formation and ensure more specific scrutiny of STS studies, there was a third stage of analysis. Here we selected the 350 top cited studies from the set of 12,045, after excluding 11 as per criteria discussed above. An analysis of the most cited studies (often 100) is a standard convention in citation studies and literature reviews in fields such as medicine, neuroscience, and its subfields (see for instance, Mendlowicz et al., 2024). We extended that here to ca. 3% of the sample (350), aiming for a more thorough search, and analysed the geographical location of leading and all authors in this set of 350 articles, especially to test how much the map of location, resulting from the 12,045 base, was reflected or substantially narrowed in this more restricted sample of top cited articles.

Finally, to perform a qualitative analysis of top publications in STS in the first 50 years of its life, we focused on the 40 most cited studies in the sample, and we also updated this value to a specific timeframe—the last decade—to check whether there was any visible shift in the geography and language of the most influential studies in the field.

## Results

### Step 1. Timeframe, geography, language, editorial policy about language submission

The 17-journal sample we ended up with is far from being homogenous or monolithic. In terms of temporal arc, it represents an interesting mix of longstanding and young journals.

Some journals were founded before the 1976 cut-off that we have used for our study: these are *Minerva* and *Social Science Information* (both 1962), and *SSS* (1971). Others were founded around the late 1980s and the 1990s, reflecting an intense growth of reflection on issues to do with science and society (*Science as Culture*, 1987; *Science and Technology Studies*, 1988; *Public Understanding of Science*, 1992; *Sociologias* and *New Genetics and Society*, 1999). Finally, others started in the new century (*Journal of Science Communication*, 2002; *Life Sciences, Society and Policy*, 2005; *BioSocieties*, 2006; *Revue d'Anthropologie des Connaissances*, 2007; *East Asian Science, Technology and Society*, 2007; *Nanoethics*, 2007; *Big Data and Society*, 2014; *Tapuya* is chronologically the latest addition [2018]). One journal, *Life Sciences, Society and Policy*, ceased publication in 2021.

### How multilingualism is promoted (or not) within the 17 journals

Seven languages of publication, for the full article and not just the abstract, were identified: English, French, German, Portuguese, Spanish, Danish, and Italian. Nearly 90% of the publications were in English. Portuguese accounted for 5%, followed by French (4.1%), and Spanish (1.0%), with Italian, Danish, and German below this threshold.

However, numbers alone do not tell us the whole story. The result of this hegemony of English could be due to the restrictive submission policies of the journals or instead individual preference of authors when a multiplicity of choices is given. For this, we analysed the official policy of the 17 journals when it comes to multilingualism. From here we grouped the journals in *four* clusters. The first has no statement about language (*BioSocieties* [Palgrave Macmillan, n.d.], *Life Sciences, Society and Policy* [now no longer active], and *Nanoethics* [Springer Nature, n.d.b]). Given that we could not find any publication in a language other than English., this cluster exemplifies a position where Englishness is taken for granted with no further problematization. Interestingly, two of these journals, *BioSocieties* and *Nanoethics*, are relatively new, a point to which we will return later to map possible trends in the field. A second cluster is explicit in mentioning that manuscripts are only accepted in English (*East Asian Science, Technology and Society, New Genetics and Society, Science as Culture* [Taylor & Francis Online, n.d.a, n.d.b, n.d.c]) or (*Minerva* [Springer Nature, n.d.a]) that submitting an article in ‘well-written English gives it its best chance for editors and reviewers to understand it and evaluate it fairly’ and offering in this case editorial assistance for improving the quality of the language. *Journal of Science Communication*—which has published several articles bilingually, English–Italian, albeit mostly in the first five years of its life—has a more qualified statement: that the abstract must always be in English, and ‘the first language of the journal is English’, with an acknowledgment that:

Papers may be submitted in other languages, but the editors reserve the right to ask for an English translation if editors or referees in the particular language are not available. If the paper can be processed in the original language and it is accepted, the journal will provide the English translation and will publish the paper in both versions (SISSA Medialab, n.d.c).

*Science and Technology Studies* (EASST, n.d.) also has a statement that indicates English is the only option: ‘A paper with a poor quality of language cannot be reviewed in an efficient and fair way, and may therefore be rejected by the editors. Authors with English as a second language should take special notice of this instruction.’ Finally, *Tapuya* (Taylor & Francis Online, n.d.d) also ‘only accept[s] and publish[es] papers in English’, though an abstract of 200 words in English, Spanish, and Portuguese must accompany the article. A paper in Spanish or Portuguese can receive feedback, but then the author ‘is responsible for the translation of the paper into English before submission’.

A third cluster is represented by the journals published by Sage (*Big Data and Society, Public Understanding of Science, Science Technology and Human Values, Social Studies of Science* [Sage Journals, n.d.a, n.d.b, n.d.c, n.d.f]) where no clear statement about a requirement for English is made. The submissions page for each journal includes identical advice for ‘authors seeking assistance with English language editing, translation etc’.^12^ Of these four Sage journals, only *Social Studies of Science* has featured non-English language articles, albeit only twice in more than four decades, reason for which it cannot be counted as multilingual journal. One interesting exception in this cluster of Sage journals is *Social Science Information* (Sage Journals, n.d.d, n.d.e). Originally founded in Paris with a bilingual title (*Social Science /Information/ sur les sciences sociales*), it stands out from the rest of the group by allowing publication in both English and French, a tradition of the journal since its pre-history as newsletter *Information* (Aymard, 2003). Here French articles reach approximately 10% of the total vs. 90% for English articles. Finally, we have a small cluster of truly multilingual journals, where multilingualism is overtly enunciated as a feature of the journal identity: *Revue d’Anthropologie des Connaissances* (*RAC*) (Open Edition Journals, n.d.), which publishes mostly in French (France/Switzerland) but allows publication in English and translates in three languages (Spanish, French, English) one key contribution per issue, and *Sociologias* (UFRGS, n.d.) in Brazil that allows publications in four languages: Spanish, Portuguese, English and French.^13^ These are the only two journals where language is clearly given a dedicated paragraph in the information to authors section, and English is allowed alongside other languages but not flagged as the preferred language. Though this sample is quite unique, it allows us to measure how English compares to other languages in a context where there is no preference for it, but an obvious connection between the journal and countries (France, Brazil) with a strong national language: in *Sociologias* (published in Brazil), English ranks third, below Portuguese and Spanish; in *RAC* (France/Switzerland) English ranks second after French. Finally, and interestingly, only two journals have attempted full multilanguage publication of whole articles (not just abstracts): *RAC* publishes trilingual articles (French, Spanish, English) for each first article of each number, since its foundation, and *Journal of Science Communication* started publishing bilingually (English, Italian), although this policy has declined over time.

**Figure 2.**
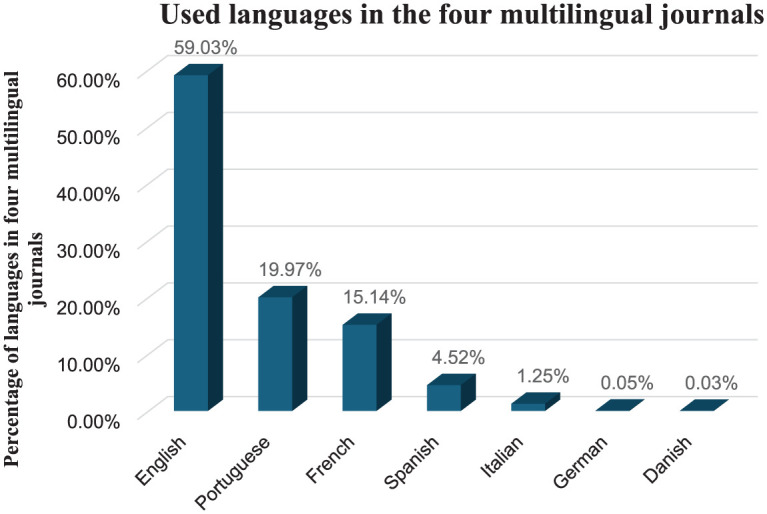
Representation of language in the four multilingual journals: *RAC, Journal of Science Communication, Sociologias, Social Science Information*.

In conclusion, only four journals can be defined as multilingual in our sample: *RAC, Journal of Science Communication, Sociologias, Social Science Information*. If we look only at the articles in these journals (and assigning fractional value to articles that were published in two or three languages at the same time), the distribution of languages is described in Figure 2, with English still predominant but less so, slightly below 60%, followed by Portuguese and French.

It is interesting to note, however, that in terms of historical trends, only *RAC* and *Sociologias* have, over time, maintained a steady balance of multilingual articles (either in the sense of one article being published in multiple languages or within the same issues), while both *Social Science Information* (although keeping a bilingual abstract) and *Journal of Science Communication* have seen a significant reduction of non-English articles after the 2010s.^14^ Interestingly, English articles have begun to appear in *Sociologias* over the past five years.

### Interplay of Geography and Multilingualism

In terms of geography of origin, two-thirds of the journals in this sample were founded and first edited by someone (leading or chief editor) based geographically in the Anglosphere’s inner core (USA, UK, Australia, New Zealand, Canada: *ST&HV, SSS, Science as Culture, NGS, BioSocieties, Minerva, PUS, Big Data and Society, Nanoethics, Life Science and Policy*),^15^ while the rest in non-English speaking countries (*Tapuya, Journal of Science Communication, RAC, EASTS, Sociologias, Science and Technology Studies, Social Science Information*): France (2), Finland, Mexico, Taiwan, and Italy. While journals like *Tapuya* and *EASTS* make a clear case for their geographical specificity, finding the location of origins for journals from the inner Anglosphere is trickier, as sometimes this is only marginally mentioned or has to be assumed from the funding body supporting the journal (like *PUS*). What is the interplay between location of the editorial office and multilingual policy of submission? Geographical location of the editorial office of course matters for the choice of language accepted in a journal, albeit in a slightly complicated way. Three patterns emerge: i) journals originating in the Anglosphere only use English as language (*Big Data and Society, ST&HV, SSS, Science as Culture, NGS, BioSocieties, Life Science Society and Policy—*with statistically insignificant exceptions, such as an article in German) even if the editorial office subsequently moves out of the Anglosphere (*Minerva, PUS, Nanoethics*); ii) journals born out of the Anglosphere in countries of romance languages (Spanish, French, Italian, Portuguese etc, evolving from Latin) display a multilingual policy although only two in a full mode (*RAC* and *Sociologias*).^16^ The only exception to this group is *Tapuya* (founded in Mexico), but much more recent than the previous group in its birth date, something to which we will return later in the conclusion; iii) two journals born out of the Anglosphere but in non-romance languages (Finland, *Science and Technology Studies* and Taiwan, *EASTS*) have opted for English as the best resource form the start (*Tapuya* also can be considered in this category). Finally, looking at the whole historical arc, the location of the journal editors is not of course something that stays necessarily stable, but changes over time. However, with few exceptions, these movements do not challenge the borders of the Anglosphere’s inner core. For instance, *ST&HV* has recently moved from the US to Australia and New Zealand. *BioSocieties* has also extended its editorial leadership to an Australia-based colleague. Others—*SSS, NGS*—have always remained stably within the Anglosphere inner core. So too have the journals within Northern Europe, that is *Science and Technology Studies* (Finland), *Nanoethics* (Germany), and *Revue d’Anthropologie de Connaissance RAC* (in France and then Switzerland). Two exceptions are *PUS* and *Minerva*, which both moved from the inner circle of the Anglosphere to Germany (*PUS* via Italy). Vice versa, only one journal founded outside the Anglosphere has currently established an Anglosphere-based editor; the *Journal of Science Communication* started in Italy and continues to be based on an Italian platform (jcom.sissa.it), but the new editor-in-chief is affiliated with the Queensland University of Technology (Australia), while the deputy editor is based with Stellenbosch University (South Africa). Of note, these are the only cases of permeability of the English/non-English divide in the selected sample.^17^

### Step 2. The 12,045 articles from the 17 journals, featuring 10,708 with affiliation data

**Figure 3.**
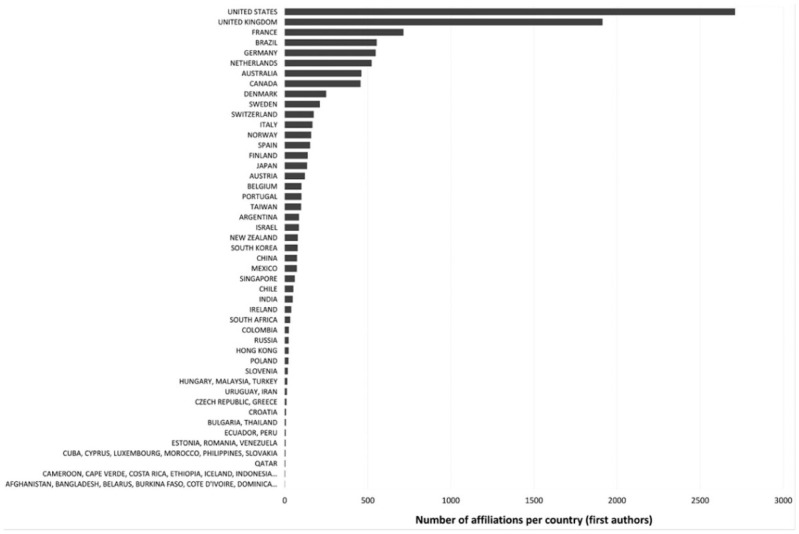
Geographic distribution of first authors in 17 top STS journals, 1976 to 2023 (n = 10,708).^20^

Of the 12,045 articles that met all inclusion criteria for analysis (see criteria in Figure 1), 10,708 included affiliation data for this analysis. Given the limitations of this sample, for this step we decided to limit our work to: a) offering a visual image of the geographical affiliations of all first authors (i.e., for whom affiliation data is available) (Figure 3), b) an overall analysis of the main countries where clusters of STS publications can be found, c) publications per country data normalized to country size and d) the evolution of these results across different decades.

In terms of overall analysis, there emerged an interesting picture documenting scholarly contributions to these journals from several STS communities not included in Euro-American clusters. Of relevance are first authors in South and Central America: specifically, from Brazil (553 contributions), Argentina (86), Mexico (73), Chile (52), and Colombia (24). Africa presents an interesting knot, with South Africa having 33 contributions, Morocco following with 4, and several English-speaking African countries represented with 1 or 2 affiliations. Asia offers a very diversified map with Japan (134), China (74) and India (48) well represented. Russia has a value of 23, Australia has 461, and New Zealand, 78. We can say that this map reflects quite consistently the economic investment of countries in technology and science and longstanding promotion (or not) of research on technological and scientific matters. First authorship from the Global South^18^ accounted for the 11.79% share in the broader corpus.

In terms of population size, only five countries (Denmark, Netherlands, Norway, the UK and Finland) achieve a ratio higher than two first-authored publications per 100,000 population (UNCTAD, n.d.b). Denmark’s high ratio (4.166) is especially noteworthy, being 1.297 higher than the Netherlands (2.869), in second place. Only 11 countries achieve a ratio higher than 1.2 at all (including Dominica, which has a single lead-authored publication and a population of only 66,205). With the second highest number of first authors overall (1910 compared with the United States’s 2708), the United Kingdom’s high impact relative to population size is demonstrated by its ratio of 2.763—comparable to the most high-ranking countries behind Denmark (4.166), Netherlands (2.869), Norway (2.851) and Finland (2.439). The United States’s ratio of 0.784 sits in 20th place. If we look at country variations over time (for three decades respectively: 1993–2002, 2003–2012, 2013–2022), there appears to be a gradual homogenization evident across STS publications. Comparing the countries with the highest 22 first author affiliations^19^ as between the decades 1993–2002 and 2003–2012, for example, five countries represented in the earlier decade (India, Israel, Mexico, Hungary and Portugal) fell outside the top 22 for the later decade (being replaced by Switzerland, New Zealand, Taiwan, Belgium and South Korea). As between the decades 2003–2012 and 2012–2022, however, there were only two differences (New Zealand and last-placed South Korea being replaced by Portugal and last-placed Israel).

### Step 3. The top cited studies: The long list of 350 and what is not in it

**Figure 4.**
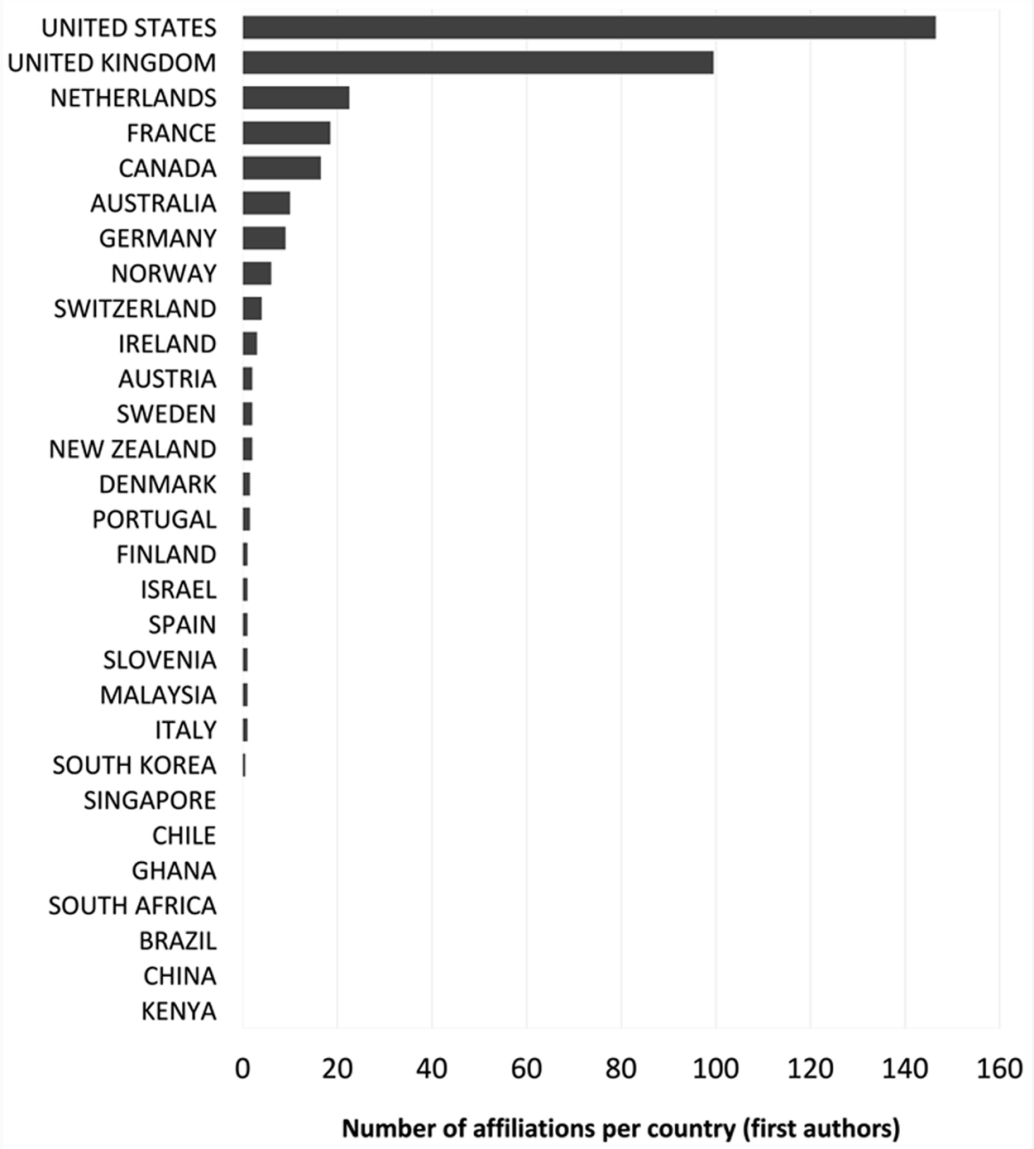
Geographic distribution of highly cited articles in 17 top STS journals, 1976 to 2023 (n = 350)—first authors.

One important limitation of the previous section of the analysis is that having proceeded by including all article-length publications for all the 17 journals, it is not possible to tell from this global view how many of these articles really engage with broadly defined topics in STS. Given that a manual analysis of the sample is unmanageable we focus on the 350 most-cited articles, approximately 3% of the overall search (see Figure 1). Here, however, we find a much more selective situation in terms of language and geography than the wider picture of the full dataset (i.e., the 10,708 articles for which Scopus provides affiliation data). In terms of language, the only article not published exclusively in English^21^ that makes the cut is Trompette and Vinck’s (2009) ‘Retour sur la notion d’objet frontière’. In terms of geography, we decided to represent all authors contributing to the top 350 and gave fractional value when one author had double affiliation in two different countries (but not in the case where they are simply listed as ‘visiting’) (Figure 4). Despite the decision not to focus only on first authors, the results are quite hierarchical, with the US and the UK largely leading with a value respectively of 147.5 and 99.5 for first author. In the first author column, only four other countries have double-digit results: Netherlands with 22.5, France with 18.5, Canada with 16.5, and Australia with 10. Notably, Germany has only 9. Southern European countries are poorly represented: Portugal has 1.5, Italy and Spain 1 each. Ranks for second author reflect a similar hierarchy, although with the UK exceeding the US (UK 47.5 and USA 45) and the rest quite far below numerically (Netherlands 3^rd^ at 15, then Canada and France at 5). Outside of Euro-America, Chile (not represented in the first author rank) has a second author in the list. South Africa and China, not represented in first and second authors, have respectively 1 and 0.5 value in third authors (guided again by the UK with 21 and USA 18). Brazil, Kenya, Ghana and Singapore appear in the list of fourth or higher authors with a value of 1. First authorship from the Global South shrinks to a mere 0.28% of the sample—a drastic reduction from nearly 12% when considering the broader corpus.

### Representation of the 17 journals in the top 350 list

How are the 17 journals represented within the sample of 350 most cited articles? Fairly unequally. The bottleneck effect operates not only in terms of geography or language but also global prestige, impact factor and reputation for respective journals. The most represented journals are: *SSS*, with 125 outputs and the top 3 ranked articles, *ST&HV*, with 77 outputs and the 4^th^, 5^th^ and 7^th^-ranked articles, and *PUS*68, with outputs the highest ranked of which is 12^th^. This group of journals represent the lion’s share of the sample. There is then a group of journals whose outputs as ranked represent a third, or less, of the top three journals: *Big Data and Society* (22 articles, with its most cited article ranked 6^th^), *Social Science Information* (15 articles,^22^ its most cited article ranking 10^th^), *BioSocieties* (9 articles, its most cited article ranking 18^th^), *Minerva* (12 articles, its most cited ranking 14^th^), and *Science as Culture* (13 articles). Finally, there is a group with fewer outputs, all ranking quite low: *Nanoethics* with 5 outputs and its best at 116^th^; *Revue d’Anthropologie des Connaissances* with 1 output at 295^th^; *Science and Technology Studies* with 1 output at 211^th^; and *Life Sciences, Society and Policy* with 2 outputs, the most highly cited at 259^th^. Importantly, five of the original 17 journals are not represented in the top 350 at all: *Journal of Science Communication, New Genetics and Society, Sociologias, East Asian Science, Technology and Society*, and *Tapuya* (the last being a much younger journal). It is worth noting that four of these five journals have editorial offices not based in the inner core of the Anglosphere.

### Step 4. Three clusters from the qualitative analysis of top 40 publications

We further focused our analysis to identify the most influential documents in the field, which can be said to constitute at least one aspect of the canon. We compiled a list of the 40 most-cited publications, which can be found in Table A1 (Appendix), a list headed by Star and Griesemer (*1989*, citations to top-40 articles in Table A1 are italicized) ‘Institutional ecology, “translations” and boundary objects’ with 6,461 citations in Scopus on the date of the search.

**Figure 5.**
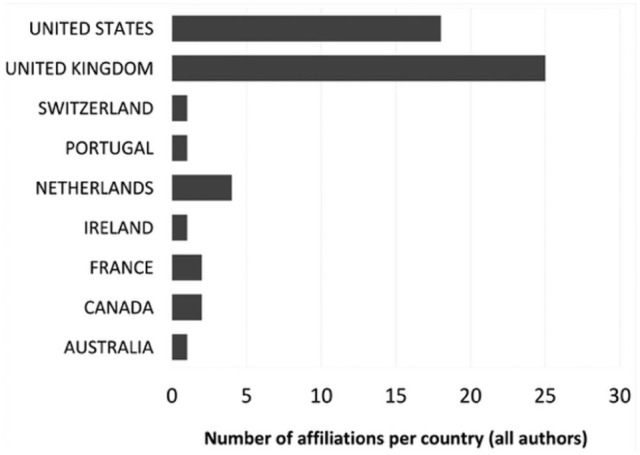
Top 40 map for all authors.

A closer analysis of the top 40 outputs in STS and STS-related journals presents several phenomena of interest (see Table A1 and Figure 5 for details). The geography of authors’ institutional affiliation appears particularly narrow. The UK and the US dominate the top 40 publication list. In proportion to its population, the UK is the single most important contributor to this top list. France and the Netherlands are the two non-Anglophone countries (Netherlands perhaps part of an expanding circle of the Anglosphere) contributing more than one article to the list, both in English.^23^ Finally, Portugal is the only country from Southern Europe with an output. Interestingly, however, the topic is a study of the British ‘prestige press’ attitude to climate change and the author (*Carvalho, 2007*) has a PhD from a UK university.^24^ In terms of qualitative analysis, the articles in the top 40 ranking can be gathered in three clusters: a) ‘placeless analysis’; b) ‘national case studies’; and c) ‘light cosmopolitanism’.

#### Placeless analysis

The first cluster presents theoretical or methodological contributions *with no clear or explicit geographical location* or empirical focus in a specific place. However, this placelessness is relative or even fictional, given that many of the cultural sources for the theoretical examples refer to specific Anglo-American scientific and technological contexts: Charles Darwin (*Kitchin, 2014*), US advocacy (*Hilgartner, 1990*), former UK PM Tony Blair’s speeches and writings on science policy (*Stirling, 2008*), British American post WWII debates on eugenics, biomedicine, and biological racism (*Rabinow & Rose, 2006*), and nudging policy from the Royal Bank of Canada (*Mittelstadt et al., 2016*). Others more explicitly deal with US or UK sources. In his ‘The politics of talk’, Irwin (Liverpool), deals with scientific governance ‘taking the British example’ or ‘building especially upon recent British experience’ (*Irwin, 2006*, p. 299); besides sources from the House of Lords, there are references to documents from the European Commission, and two other European countries are mentioned in passing, Denmark and the Netherlands (p. 299). Rowe and Frewer’s article on ‘A typology of public engagement mechanisms’ is a conceptual article with a focus on a classification of public participation mechanisms. Key sources, however, are localized, mostly in the UK and US (see *Rowe & Frewer, 2004*, p. 257). As the authors themselves recognize: ‘Although there are more than 100 mechanisms listed, the bias is on UK and US types.’ It is hence recognized that ‘in other countries, these particular mechanisms [of public participation] may be known by different names, or there may exist still other mechanisms’ (*Rowe & Frewer, 2004*, p. 256). This insight, however, is not further developed in the article. Stirling’s (*2008*) article on ‘Power, participation, and pluralism in the social appraisal of technology’ is also theoretical but examples are drawn from UK politics and science policy on nuclear power. There are, however, a couple of passing references to more international institutions such as the United Nations Development Programme 2000 or the Commission of the European Communities. Further, ‘Surveillance, Snowden, and Big Data’ by Lyon (*2014*), based at Queen’s University in Canada, follows the 2013 Snowden’s case and reflects on the contemporary dilemma of Big Data and surveillance. Albeit theoretical, its geographical underpinning is also narrow, mostly focusing on a US collection of metadata after 9/11 and adding that ‘it is unclear how far similar such programs extend to *other countries such as Canada or the UK*’ (*Lyon, 2014*, p. 3, our italics). Collins and Evans’ (*2002*) ‘Third Wave of Science Studies’ has also specific localized references to case studies about AIDS treatment in San Francisco or British Electric companies in Leicestershire, England, and one reference to the EU white paper on science governance. This is not to say that all these authors take deliberately their local case as a synonym for the universal, but the neglect of information outside their narrow geographical focus has, in the end, a cumulative universalizing effect.

#### National case studies

A second cluster is not placeless but based on the empirical analysis of a specific location. One case in point is the most cited output in the table, Star’s and Griesemer’s (*1989*) article on ‘boundary objects’, since then a key concept in STS work. Both authors based in California at the time (Irvine and Davis), the concept has a specific empirical location of source, which is the Museum of Vertebrate Zoology (MVZ) at the University of California, Berkeley. Also, Star’s (*2010*) second article in the sample (ranking 5th), although theoretical, reflects a Californian empirical set of data, such as archival work at the Bancroft Library at the University of California, Berkeley. Ranking 12^th^, Wynne’s (1992) ‘Misunderstood misunderstanding: Social identities and public uptake of science’ is a case study, from Northern England, of sheep farmers’ responses to the restrictions introduced after Chernobyl’s nuclear disaster. Epstein’s (*1995*) ‘The construction of lay expertise: AIDS activism and the forging of credibility in the reform of clinical trials’ is similar; based at the University of California San Diego, Epstein’s article focuses on AIDS activism in the United States, drawing on interviews in San Francisco and, more widely, the US. The author recognizes in a few passages the tension between their localized analysis and possible more global trends on the development of health-related activism. They speak of ‘influence that appears to be exerting, *at least in* the United States, on a new wave of health-related activism’ (p. 410, our italics). Similarly, Lee and Bozeman (*2005*) discuss ‘The impact of research collaboration on scientific productivity’ analysing survey responses and CVs of more than 400 academic scientists working in USA universities or research centres (see similarly *Cummings & Kiesler, 2005*). Ranking 13^th^, Fiorino (*1990*) is an author based at the US Environmental Protection Agency. His article offers a survey of five institutional mechanisms for allowing the lay public to influence environmental risk decisions. Unlike previous articles, however, here it is not noted that this is a national case study of specific participatory mechanisms, thereby slipping more easily into a transhistorical and transnational kind of claim that is viewed from nowhere. Finally, Jasanoff’s (*2003*) ‘Technologies of humility’ is also largely a US-centric article, with a few non-US references going to the German sociologist Ulrich Beck and the House of Lords Select Committee on Science and Technology in Britain. Authors in these clusters may have problematized at different levels the possible generalization of their own specific and local—or national, at best—findings. However, given that all the articles reflect the Anglosphere inner circle, this raises the issue whether this group of leading articles in the field may not just be embedded in a very specific linguistic framework, but also that the empirical and epistemological grids from which universal concepts are made may have a very clearly defined location.

#### Light cosmopolitanism

Finally, a third cluster deals with the issue of linguistic and theoretical ‘translation’ or comparison, assuming explicitly the problem of a multiplicity of languages, worldviews, and theoretical repertoires that may be in tension among them. Callon et al. (*1983*), for instance, focus on issues of nutrition science translation between French and US or UK research centres. Jasanoff and Kim’s (*2009*) ‘Containing the atom’ develops its notion of sociotechnical imaginary from a two-country comparison of nuclear power in the US and South Korea; both authors were based at Harvard at the time. In the bibliography, it refers to more than 20 original sources in Korean (audits, documents from the Korea Nuclear Energy Foundation or National Assembly, etc.). Another article dealing with issues of translation is Mol and Law’s (*1994*) ‘Regions, networks and fluids: Anaemia and social topology’. Based on Dutch sources including interviews with Dutch tropical doctors, the authors recognize that in some cases there might be tensions in moving from one linguistic repertoire to another: They claim that ‘finding an English equivalent for a somewhat strange Dutch expression is often extremely hard’ (*Mol & Law, 1994*, p. 665). Given that this is one of the few articles in the sample dealing explicitly with the Global South, it is interesting to note, however, that the mobility of the concept of anemia across networks occurs ‘between the Netherlands and Africa and back’ (*Mol & Law, 1994*, p. 664). That is ‘Africa’, a broad toponym that is used to capture different national contexts in the African continent and that runs the risk of making amorphous or homogeneous the specificities across individual countries: this trope occurs in several recurring passages, another example is a reference to ‘a factory in Germany or Korea to the Netherlands and Africa’ (*Mol & Law, 1994*, p. 649). Bauer et al. (*2007*, p. 82) mention in passing audits of scientific literacy in ‘US, Canada, China, Brazil, India, Korea, Japan, Bulgaria, Switzerland, Singapore, Britain, Germany and France and many other EU countries’, and have a UK-Portugal comparison on science and the public, with a reference in the Portuguese language (da Costa et al., 2002, *Publicos da ciencia em Portugal*). However, the US and the UK remain largely as benchmarks and key sources for a theoretical agenda on the public understanding of science (see also *Etzkowitz, 2003*, for light cosmopolitanism).

Overall, across the three clusters—except for the figures in French in Callon et al. (*1983*)—non-English words appear only a few times in the main texts of this corpus. It is the case, for instance, of the African word *nganga* (a local water diviner) in de Laet and Mol (*2000*), and the German *Dingpolitik* and the Latin *cura* in Puig de la Bellacasa (*2011*). It would be wrong to say that these terms are used in a tokenistic way across the three clusters, nor do we wish to overlook how these fellow STS scholars have, in multiple ways, addressed these questions (see, for instance, Mol, 2025). Some contributors to the top 40 have authored elsewhere publications in languages other than English (for instance Godin and Bauer, French). Yet, taken together the three clusters illustrate how there is little context or engagement with possible theoretical alterity embedded in influential scholarship and conceptual work in STS. Across the three clusters, most of the intellectual references are to British and American sociological theory. There is a consistent canon of Dutch, French, German and Italian authors, mostly anthropologists (van Gennep, see *Crouch & McKenzie, 2006*), philosophers (Gadamer, Habermas) or social theorists (Weber, Marx, Foucault, Negri, Agamben), that are referenced mostly in the English translation, but sometimes in French or Italian original (e.g. *Rabinow & Rose, 2006*). Non-Anglo-American sources however rarely exceed 10% of the overall reference list. Based in a French institution, the article by Fischler is an exception: approximately 50% or more of references are given in the original French. de Laet and Mol (2000) on Zimbabwe have referenced some significant Dutch technical literature, Puig de la Bellacasa (*2011*) has four references in Spanish, and Marres (*2007*) has French (1), Dutch (2), and German (2) references. Finally, in terms of journal’s representation, in the 40 most cited articles, only seven journals out of seventeen are represented: *SSS* with 13 outputs, *ST&HV* with 10, *PUS* with 5, *Big Data and Society* and *Social Science Information* with 4 each, *Minerva* with 3, and *BioSocieties* with 1.

### Top 10 outputs from the last decade (2014-2023)

To assess if this situation is moving in a dynamic way toward greater pluralization, we also analysed the top 10 outputs of the last decade (2014-2023) removing the 3 already included in the top 30 to avoid duplication. The major shift is not in language or geography but the substantial rise in outputs from a journal like *Big Data and Society* (current editor-in-chief in the USA, managing editor in the UK) which has 6 outputs in the top ten. However, if we remove the 4 outputs (all from *Big Data and Society*) already in the top 30, we obtain the following results. *Big Data and Society*, 3 outputs, country of affiliation US (2) and Ireland (1); *Social Studies of Science*, 3 outputs, country of affiliation US (2), and UK (1); *Science Technology & Human Values*, 1 output, US; *Public Understanding of Science*, 3 outputs with country of affiliation in the US (2) and UK (1). At least for this very specific sampling of the most cited publications, admittedly quite small in number, it is interesting to remark however that geography and language do not show any particular dynamism, nor do they reflect wider debates and commitments across the global STS community out of Euro-America.

## Conclusions

Born out of a context that still reflected the ideological and geopolitical tensions of the 1960s and the 1970s, including the Cold War, and a polarization of the world between the West and the Rest (Aronova & Turchetti, 2016; MacLeod, 2016), over the years, STS has visibly expanded its epistemic and geographic horizons. Recently, STS scholars have increasingly engaged with debates on citational justice, epistemic decentralization, and alternative infrastructures for knowledge dissemination. For example, recent responsible research and innovation (RRI) projects explicitly incorporate indigenous knowledges and community-based research, affirming the value of epistemic pluralism beyond academic silos (Zwart et al., 2024). These efforts are not marginal: They signal a growing institutional recognition that epistemic inclusion is crucial to the future vitality of the field. Despite these important gestures toward pluralization, our bibliometric and qualitative analyses reveal enduring asymmetries that reflect both historical sedimentations and ongoing structural exclusions, particularly when examining the most cited and hence ‘exemplary’ or ‘canonical’ outputs.

The overview of the 17 journals showed that while seven languages were nominally present, English accounted for an overwhelming majority of publications, with Portuguese, French, Spanish, Italian, German and Danish trailing far behind. Moreover, language policy and editorial geography revealed telling patterns: journals based in the Anglosphere predominantly enforce English-only policies, while multilingual journals are largely situated outside these centers—yet remain underrepresented in the citation landscape.

In the second stage, examining 12,045 articles, we saw a larger breadth of geographic affiliation, with notable activity from countries outside Euro-North American institutions, such as Brazil, Argentina, Japan, and India. This speaks to an encouraging globalization of STS research, perhaps driven by massive global investments in technoscience in the same regions of the world. However, when we narrowed our focus to the 350 most-cited articles (stage three), these patterns collapsed into a far more concentrated map. Authorship from the US and the UK dominated, with only marginal representation from the Global South. Further focusing our analysis on the 40 most cited publications underscored this bottleneck effect, with the UK and US largely leading the rank. Taken together, these findings align with Kreimer’s (2022a, 2022b) observation that while STS has always aspired to emphasize the local and situated character of knowledge production, it replicates the very asymmetries it seeks to critique. Similarly, as Singh and Lynch (2025) demonstrate, the discipline’s proverbial economies circulate familiar cases and canonical references, often at the expense of less visible but equally rich STS traditions developing outside core Euro-North American institutions.

To this end, we ask: What should we do next? How do we pluralize the voices, languages, and epistemologies reflected in STS? And how do we undo, collectively, the bottleneck effect producing an asymmetrical canonical corpus of the discipline? We believe our findings call for a deliberate and collective strategy to reimagine the intellectual and infrastructural future of STS. This requires action on multiple fronts, including a sustained attention to symmetry (Law & Lin, 2017). This *internalized symmetry*, we argue, urges us to move beyond self- or partial-reference and open our critical vocabulary to more diverse epistemic traditions. It encourages STS scholarship to further multiply voices and experiences by threading the life of concepts beyond the Anglosphere. This ‘opening up’ (Stirling, 2007) is, after all, what made STS attractive for many of us: pluralizing the worlds of Science, written with capital S, diffracting this ‘singular’ and ‘capitalized’ fiction through the actual networks, assemblages, and microforces of knowledge production. In the remaining, we briefly elaborate upon two ways to internalize symmetry in STS: citational justice and institutional inclusion.

First, and building on Singh and Lynch’s (2025) suggestion, STS scholars should carefully attend to the implicit intellectual hegemony of their discipline thriving on proverbial economies—that is, familiar, widely-circulated stories and references that both stabilize and delimit our field . Our findings call for moving beyond passive recitation of the discipline’s idioms towards a symmetrical re-distribution of citational credit. Concrete steps include: systematically diversifying bibliographies, foregrounding works in multiple languages or identifying gaps, and valuing conceptual contributions from marginalized epistemic locations. STS scholars should also critically reflect on the ‘usual suspects’ in canonical references and actively seek less-cited, yet no less insightful, interlocutors.

Second, we wish to reiterate here Rodriguez Medina’s (2024) (and related) crucial reminder: Epistemic decentralization is not merely about intellectual pluralism (or decentering thinking from hegemonic institutions) but about decentralizing the material infrastructures of production and recognition. STS scholars can build and contribute to platforms of collaboration outside traditional academic hubs—for example, regional journals, open-access repositories, or conference networks. STS associations and journal editors can play a pivotal role here by rethinking peer review processes, supporting translation initiatives, recognizing outputs published in languages other than English, and fostering participation in editorial boards from scholars across multiple regions and languages, or even moving editorial boards on a rotation principle out of the Global North (or combining at the same period a Global North with a Global South editorial location). The goal is to create ecosystems where scholars from outside dominant institutions are not mere participants or spectators in ‘global’ STS conversations, but rather co-architects of the field’s intellectual agenda. As we have evidenced in the first pages, our article does not fall into a vacuum but comes at a moment where a significant critical rethinking of what is canonical is occurring in many disciplines, and vibrant initiatives aiming at pluralizing STS are pursued by a multitude of voices. In short, the future of inclusion and intellectual diversity in STS depends on whether these voices will multiply and enable the field to enact the very principles of pluralism, attention to local processes, and symmetry it so often champions. Rather than treating these as add-on concerns, or merely as matters of linguistic diversity, we propose internalizing them at the heart of STS’s intellectual and institutional practices. Only by doing so can the field genuinely realize its promise of becoming a global epistemic endeavor. However, as always, we need to balance hope with realism, and remain aware that there is still a huge amount of work of generosity, care, and courage to think outisde consolidated networks of knowledge/power, if we want to nurture a new generation of genuinely post-Euro/American-centric scholars. They, in the future, may look at the language of ‘placeless analysis’, ‘national studies’ (taking Euroamerica for granted), and ‘light cosmopolitanism’ as something from a distant past: just as the first generation of STS scholars looked at canonical and established science as something requiring a radical restructuring to make it dynamic, contingent, and plural.

## Appendix

**Table A1.** Top 40 Articles.

Rank	Authors	Year	Journal	Cited by (Scopus)	No of years published (footnote 8 for clarification)
1	Star and Griesemer	1989	SSS	6461	34
2	Collins and Evans	2002	SSS	1486	20*
3	Pinch and Bijker	1984	SSS	1479	39
4	Rowe and Frewer	2000	ST&HV	1396	22*
5	Star	2010	ST&HV	1297	13
6	Kitchin	2014	Big Data & Soc.	1222	9
7	Rowe and Frewer	2005	ST&HV	1174	18
8	Lee and Bozeman	2005	SSS	1041	17*
9	Stirling	2008	ST&HV	1039	15
10	Callon et al.	1983	Soc. Sci. Inf.	997	40
11	Burrell	2016	Big Data & Soc.	960	7
12	Wynne	1992	PUS	955	31
13	Fiorino	1990	ST&HV	902	33
14	Jasanoff and Kim	2009	Minerva	901	14
15	Jasanoff	2003	Minerva	878	19*
16	Crouch and Mckenzie	2006	Soc. Sci. Inf.	876	17
17	Etzkowitz	2003	Soc. Sci. Inf.	808	19*
18	Rabinow and Rose	2006	BioSocieties	806	17
19	Nowotny, Scott and Gibbons	2003	Minerva	798	19*
20	Fischler	1988	Soc. Sci. Inf.	798	35
21	Mol and Law	1994	SSS	790	29
22	de Laet and Mol	2000	SSS	785	23
23	Mittelstadt et al.	2016	Big Data & Soc.	767	7
24	Sturgis and Allum	2004	PUS	760	19
25	Epstein	1995	ST&HV	678	28
26	Puig de la Bellacasa	2011	SSS	668	12
27	Jasanoff	1987	SSS	589	36
28	Rowe and Frewer	2004	ST&HV	571	18*
29	Godin	2006	ST&HV	557	16*
30	Irwin	2006	SSS	557	17
31	Irwin	2001	PUS	531	22
32	Winner	1993	ST&HV	505	30
33	Hilgartner	1990	SSS	504	33
34	Wynne	1991	ST&HV	502	32
35	Carvalho	2007	PUS	490	16
36	Bauer et al.	2007	PUS	488	15*
37	Marres	2007	SSS	488	16
38	Cummings and Kiesler	2005	SSS	470	17*
39	Lyon	2014	Big Data & Soc.	463	9
40	Gilbert	1977	SSS	456	46

## Notes

1. There are many possible *terminus a quo* for STS, and the boundaries and meanings of the phrase are debated and ambiguous. While, heuristically, the first issue of *Science Studies* in 1971 was one potential moment when the field began to develop certain norms (‘Editorial’, 1971), and the foundation in 1975 of The Society for Social Studies of Science (4S) as an international, nonprofit association could be considered a starting point, we took instead the year of foundation of the *Newsletter on Science and Technology & Human Values* (1976), rebranded *Science, Technology, & Human Values* in 1978. The *Newsletter* was founded with the financial support of the National Science Foundation and the National Endowment for the Humanities and produced under the auspices of the Harvard University Program on Science. The first issue appeared in October 1976, which is at the beginning of the academic year 1976-77, but for reasons of simplicity in our search we selected 1 January 1976 as start date (see Method section).

2. While citations are not necessarily a direct index of a given article’s place within the canon, highly cited articles are more likely to translate into substantial and thus influence the works that reference it (Teplitskiy et al., 2022). As Indigenous, feminist and Black scholars have repeatedly pointed out, the politics of citation offers a powerful glimpse into elements of canon formation (Ahmed, 2013; Hovland, 2024; Kim, 2020; Liboiron et al., 2017; C. Smith, et al., 2021).

3. Incidentally the English word *cannon* is also related to the Greek κάννα and Latin *canna* (via the French *canon and the Italian cannone*). The complex relationship between *canon* and *cannon*, that is between authoritative knowledge and epistemic violence, however, exceeds the scope of this article (see Meloni, in press for a wider historical treatment).

4. This feature is based on a score described broadly as follows: ‘Similarity is expressed as a percentage of referenced publications shared between the selected journals and similar ones in descending order’ (Scimago, 2024).

5. In terms of language, Scopus does include non-English content, however on the condition that an English title and abstract are made available (Scopus, 2025).

6. From what can be inferred from the metrics used at Scimago (we contacted the database editorial office on 1 July 2024 but we obtained no response) the reason why a journal like *Tapuya* (founded and covered since 2018) with a lifespan similar to *Tecnoscienza* and *Redes* (both with Scimago coverage from 2019) is included in the proximity search but not *Tecnoscienza* and *Redes* is that these latter journals have not generated enough data to be captured by the Scimago algorithm in the proximity search (from a manual search conducted with Deakin University Library staff at their university on 3 July 2024, *Tapuya* has ca. 2.5 times the number of articles and hence references, which are used to generate the metrics of the proximity search, of the other two journals).

7. While this 1.7% might be considered a reasonable margin of error, across a corpus of over 94 million records, it is a sizeable number. There is also good reason to suspect that the errors may disproportionately affect non-English sources, particularly as a result of incorrect spelling generated by language specific characters (diacritics, accents) (Pranckutė, 2021).

8. To incorporate these originally wrongly classified ‘Review’ articles into our dataset, we used the Scopus ‘Citation overview’ feature to export only citations that had occurred up to and including 2022 (i.e., to 12 December 2022) as it was not possible to obtain data for our original cut-off date of 15th June 2023. This minor 6 month difference in data is reflected in the number of years from publication in Table 1, last column (years with asterisk), but has no impact on the overall results.

9. We excluded three articles by Pierre Bourdieu, an author who could have been incorporated into the STS canon, because they did not directly address science and technology, but areas such as sport, genesis of groups, and linguistic exchange.

10. Other scholars for instance in the sociology and anthropology of work, networks, finance or organizations have certainly contributed to STS core readings but are not reflected in our corpus having published largely outside the selected 17 journals. Similarly, many debates in different disciplines (organization studies, workplace studies, communication, and media studies) have been shaped by STS approaches but would not appear in our search. We are grateful to Dominique Vinck for this valuable point.

11. Resolved to a scale of 0 [no similarity] to 100 [maximum similarity]: SCIMago contact office, personal communication, email with Sam Cadman, on 26 March 2025.

12. The advice includes a link to the paid service, Sage Author Services (n.d.a). One of the offered services is advertised as ‘English Language Editing’ (Sage Author Services, n.d.b): ‘[editing and formatting] by subject matter experts who provide you an error-free and grammatically correct manuscript, along with feedback and suggestions on your writing, thereby delivering a document meeting the international standards of publication’.

13. The prioritization of the languages other than English in *RAC* and *Sociologias* stands in stark contrast to the other journals in our sample. The former emphasizes that it is a ‘French-language journal’ that however, it also offers the possibility for authors “to publish a version in other languages of texts accepted for publication in French” (Open Edition Journals, n.d, translation by first author). The latter accepts “submissions in Portuguese, Spanish, French or English” and if an article in English or French is accepted it “must be translated into Portuguese and published in both languages.” (https://seer.ufrgs.br/index.php/sociologias/information/authors, Accessed 21 Sept 2025, translation by first author). In both cases authors are responsible for their translation, with *Sociologias* available to offer a list of potential translators.

14. However, the *Journal of Science Communication* has branched out since 2018 into a second ‘regional’ journal, *JCOM América Latina (JCOMAL)*, which was not identified in our Scimago proximity search. The journal has published in Portuguese and Spanish, consistently since 2022 with abstracts and titles in English. The editor in chief, Luisa Massarani, is based in Brazil, at the Instituto Nacional de Comunicação Pública da Ciência e Tecnologia e Fundação Oswaldo Cruz (SISSA Medialab, n.d.b). The website retains a ‘.it’ address, reflecting the foundation in Italy of the parent journal.

15. For the foundation and history of the term Anglosphere see Vucetic (2008). We speak here of the Anglosphere Inner Core loosely following a linguistic convention established firstly in Kachru et al. (1985) with reference to World Englishes. For an update on the history of the term see Schmitz (2014). A non-dissimilar model but in four level (core-middle-outer-periphery) is highlighted in Bennett (2015).

16. *Journal of Science Communication* (current editor in Australia) has published in the past in Italian, and French/Spanish but was at the time located, in terms of editorial office, out of the Anglosphere, in Trieste, Italy. (SISSA Medialab, n.d.a).

17. *Life Sciences, Society and Policy* was founded in 2005 as *Genomics, Society and Policy*, within the ESRC Genomics Network in the UK but in a partnership with the Dutch Centre for Society and Genomics. The first editor Ruth Chadwick was based in the UK.

18. Using UNCTAD’s classification of “developing” economies (UNCTAD, n.d.a). See for correspondence to North/South terminology, UNCTAD (2022, p. 21).

19. For the decade ending 2002, the four countries ranked in the 19th to 22nd places (Argentina, Denmark, Hungary, and Portugal) all had five first author affiliations each.

20. Figure 3 shows the first author affiliation countries in the full dataset (n = 10,708), from the United States (highest with 2,710) down to countries with one first author affiliation each. Where multiple countries have the same number of first-authored articles (and that number is fewer than 20), the countries are alphabetized and combined for ease of representation (e.g., Hungary, Malaysia and Turkey all have 15 first-authored articles each). For countries with two first-authored articles or less, the lists above have been truncated.

21. As it was published in three languages, French (originally), English, and Spanish, it is impossible to track the distribution of Scopus citations across the three languages.

22. It must be noted that *Social Science Information* had 11 articles excluded from the top 350 list based on the exclusion criteria previously explained (articles not specifically discussing *science and society*, but either one or the other).

23. By counting authors and coauthors, France with 2 outputs, Netherlands with 4.

24. We made use of this information when it was available on the published article itself.

## References

[bibr1-03063127251386080] Ahmed S. (2013, September 11). Making feminist points. feministkilljoys. https://feministkilljoys.com/2013/09/11/making-feminist-points/

[bibr2-03063127251386080] Alatas S. F. (2022). Knowledge hegemonies and autonomous knowledge. Third World Quarterly 1–18. 10.1080/01436597.2022.2124155

[bibr3-03063127251386080] Al-Hardan A. (2018). The sociological canon reconfigured: Empire, colonial critique, and contemporary sociology. International Sociology, 33(5), 545–557. 10.1177/0268580918791967

[bibr4-03063127251386080] Ali S. Bano S. (2021). Visualization of journal ranking using Scimago: An analytical tool. Library Philosophy and Practice, 1, 1–12.

[bibr5-03063127251386080] Alkan A. Kaşdoğan D. Erol M. (2023). Placing STS in and through Turkey. Engaging Science, Technology, and Society, 9(1), 104–124.

[bibr6-03063127251386080] Amrute S. Murillo L. F. R. (2020). Introduction: Computing in/from the South. Catalyst: Feminism, Theory, Technoscience, 6(2), 1–23.37143900

[bibr7-03063127251386080] Anderson W. (2002). Introduction: Postcolonial technoscience. Social Studies of Science, 32(5/6), 643–658.

[bibr8-03063127251386080] Anderson W. (2012). Asia as method in science and technology studies. East Asian Science, Technology and Society, 6(4), 445–451. 10.1215/18752160-1572849

[bibr9-03063127251386080] Anderson W. Adams V. (2007). Pramoedya’s chickens: Postcolonial studies of technoscience. In Hackett E. J. Clarke A. E. Amsterdamska O. Lynch M. E. Wajcman J. (Eds.), The handbook of science and technology studies (pp. 181–204). MIT Press. http://ebookcentral.proquest.com/lib/unimelb/detail.action?docID=3338749

[bibr10-03063127251386080] Aronova E. Turchetti S. (2016). Science studies during the cold war and beyond. Palgrave Macmillan.

[bibr11-03063127251386080] Aymard M. (2003). In Memoriam Clemens Heller (1917-2002). Social Science Information, 42(3), 283–287. 10.1177/05390184030423001

[bibr12-03063127251386080] Baas J. Schotten M. Plume A. Côté G. Karimi R. (2020). Scopus as a curated, high-quality bibliometric data source for academic research in quantitative science studies. Quantitative Science Studies, 1(1), 377–386.

[bibr13-03063127251386080] Baber Z. (2003). Provincial universalism: The landscape of knowledge production in an era of globalization. Current Sociology, 51(6), 615–623.

[bibr14-03063127251386080] Beigel F. (2014). Publishing from the periphery: Structural heterogeneity and segmented circuits. The evaluation of scientific publications for tenure in Argentina’s CONICET. Current Sociology, 62(5), 743–765. 10.1177/0011392114533977

[bibr15-03063127251386080] Beigel F. Gallardo O. (2021). Productividad, bibliodiversidad y bilingüismo en un corpus completo de producciones científicas [Productivity, bibliodiversity, and bilingualism in a whole corpus of scientific productions]. Revista Iberoamericana de Ciencia, Tecnología y Sociedad —CTS, 16(46), 41–71. http://www.revistacts.net/wp-content/uploads/2021/03/02Beigel.pdf

[bibr16-03063127251386080] Bennett K. (2015). Towards an epistemological monoculture: Mechanisms of epistemicide in European research publication. In Alastrué R. P. Pérez-Llantada C. (Eds.), English as a scientific and research language: Debates and discourses (pp. 9–36). De Gruyter.

[bibr17-03063127251386080] Bloom H. (1994). The western canon: The books and school of the ages. Houghton Mifflin Harcourt.

[bibr18-03063127251386080] Bloor D. (1976). Knowledge and social imagery. Routledge & Kegan.

[bibr19-03063127251386080] Callon M. (1984). Some elements of a sociology of translation: Domestication of the scallops and the fishermen of St Brieuc Bay. The Sociological Review, 32(1), 196–233.

[bibr20-03063127251386080] Collyer F. M. (2018). Global patterns in the publishing of academic knowledge: Global North, global South. Current Sociology, 66(1), 56–73.

[bibr21-03063127251386080] Connell R. (1997). Why is classical theory classical? American Journal of Sociology, 102(6), 1511–1557.

[bibr22-03063127251386080] Connell R. (2019). Canons and colonies: The global trajectory of sociology. Estudos Históricos (Rio de Janeiro), 32, 349–367.

[bibr23-03063127251386080] da Costa A. F. Ávila P. D. Mateus S. (2002). Públicos da ciência em Portugal. ISCTE.

[bibr24-03063127251386080] De Laet M. Mol A . (2000). The Zimbabwe bush pump: Mechanics of a fluid technology. Social studies of science, 30(2), 225–263.

[bibr25-03063127251386080] DORA. (2012). San Francisco declaration on research assess. Retrieved October 5, 2025, from https://sfdora.org

[bibr26-03063127251386080] DORA. (2021). San Francisco declaration on research assessment. Declaration on Research Assessment. https://sfdora.org/read/

[bibr27-03063127251386080] Dumoulin Kevran D. Kleiche-Dray M. Quet M. (2018). Going South. How STS could think science in and with the South? Tapuya: Latin American Science, Technology and Society, 1(1), 280–305.

[bibr28-03063127251386080] EASST. (n.d.). Science & Technology Studies – Submissions. Retrieved September 15, 2025, from https://sciencetechnologystudies.journal.fi/about/submissions

[bibr29-03063127251386080] ‘Editorial’. (1971). Science Studies, 1(1), 1–2. 10.1177/030631277100100101

[bibr30-03063127251386080] Ehrmann J. (2022). Within, beyond or against the canon: What does it mean to decolonize social and political theory? Journal of Classical Sociology, 22(4), 388–395.

[bibr31-03063127251386080] Faruk M. Rahman M. Hasan S. (2021). How digital marketing evolved over time: A bibliometric analysis on Scopus database. Heliyon, 7(12), Article e08603. 10.1016/j.heliyon.2021.e08603 PMC8695267 34988311

[bibr32-03063127251386080] Fischer M. J. (2016). Anthropological STS in Asia. Annual Review of Anthropology, 45(1), 181–198. 10.1146/annurev-anthro-102215-100258

[bibr33-03063127251386080] Fortun K. (2018). STS across borders in brief. STS Infrastructures, Platform for Experimental Collaborative Ethnography. https://stsinfrastructures.org/content/sts-across-borders-brief

[bibr34-03063127251386080] Foster L. A. (2016). Decolonizing patent law: Postcolonial technoscience and indigenous knowledge in South Africa. Feminist Formations, 28(3), 148–173.

[bibr35-03063127251386080] Fu D. (2007). How far can East Asian STS go? A position paper. East Asian Science, Technology and Society: An International Journal, 1(1), 1–14.

[bibr36-03063127251386080] Fu D. (2020). A genealogical explication on the emergence and constructions of STS: A view from East Asia. Tapuya: Latin American Science, Technology and Society, 3(1), 192–204. 10.1080/25729861.2020.1785114

[bibr37-03063127251386080] Fuller S. (2016). Embrace the inner fox: Post-truth as the STS symmetry principle universalized. Social Epistemology Review and Reply Collective. Retrieved October 5, 2025, from https://social-epistemology.com/2016/12/25/embrace-the-inner-fox-post-truth-as-the-sts-symmetry-principle-universalized-steve-fuller/#comments

[bibr38-03063127251386080] Galassi J. (2019). Discussing the symmetry principle: Towards a realist dialogue inside global STS theory. Tapuya: Latin American Science, Technology and Society, 2(1), 32–41.

[bibr39-03063127251386080] Gordin M. D. (2015). Scientific Babel: How science was done before and after global English. University of Chicago Press.

[bibr40-03063127251386080] Grüning B. Santoro M. (2021). Is there a canon in this class? International Review of Sociology, 31(1), 7–25.

[bibr41-03063127251386080] Guerrero-Bote P. Moya-Anegón F. (2012). A further step forward in measuring journals’ scientific prestige: The SJR2 indicator. Journal of Informetrics, 6(4), 674–688.

[bibr42-03063127251386080] Harding S. G. (1998). Is science multicultural? Postcolonialisms, feminisms, and epistemologies. Race, Gender, and Science. Indiana University Press.

[bibr43-03063127251386080] Hountondji P. J. (2002). Struggle for meaning: Reflections on philosophy, culture, and democracy in Africa ( Conteh-Morgan J. , Trans.). Ohio University Press.

[bibr44-03063127251386080] Hovland I. (2024). Feminist cites: A review of feminist relations to and citations of the canon. Studies in Religion/Sciences Religieuses, 53(2), 207–226.

[bibr45-03063127251386080] Imanishi K. Asquith P. J. Kawakatsu H. Yagi S. Takasaki H. (2002). A Japanese view of nature: The world of living things by Kinji Imanishi. Taylor & Francis Group.

[bibr46-03063127251386080] Invernizzi N. Davyt A. Kreimer P. R. Rodriguez Medina L. (2022). STS between centers and peripheries: How transnational are leading STS journals? Engaging Science, Technology, and Society, 8(3), 31–62. 10.17351/ests2022.1005

[bibr47-03063127251386080] Issar S. (2025). From paradigms to pluralities: A comprehensive review of science and technology studies. Communication Research Trends, 44(2), 4–28.

[bibr48-03063127251386080] Jasanoff S. (2017). A field of its own: The emergence of science and technology studies. In Frodeman R. (Ed.), The Oxford handbook of interdisciplinarity (2nd ed., pp. 173–187). Oxford Academic. 10.1093/oxfordhb/9780198733522.013.15

[bibr49-03063127251386080] Kachru B. B. Quirk R. Widdowson H. G. (1985). Standards, codification and sociolinguistic realism. In Bolton K. Kachru B. B. (Eds.), World Englishes: Critical concepts in linguistics (pp. 241–270). Routledge.

[bibr50-03063127251386080] Kaltenbrunner W. Birch K. Van Leeuwen T. Amuchastegui M. (2022). Changing publication practices and the typification of the journal article in science and technology studies. Social Studies of Science, 52(5), 758–782. 10.1177/03063127221110623 PMC9483190 35903817

[bibr51-03063127251386080] Kaşdoğan D. Fortun K. (2020). About the transnational STS network. STS Infrastructures, Platform for Experimental Collaborative Ethnography. https://stsinfrastructures.org/content/about-transnational-sts-network

[bibr52-03063127251386080] Kennedy G. (2013). The origin of the concept of a canon and its application to the Greek and Latin classics. In Gorak J. (Ed.), Canon vs. culture (pp. 105–116). Routledge.

[bibr53-03063127251386080] Kim A. L. (2020). The politics of citation. Diacritics, 48(3), 4–9. 10.1353/dia.2020.0016

[bibr54-03063127251386080] Klein S. Toledo Ferreira M. (2024). Lélia Gonzalez: An Amefrican perspective to reorient the canon. Tapuya: Latin American Science, Technology and Society, 7(1), Article 2359840.

[bibr55-03063127251386080] Kondo S. Swanson H. A. (2020). 鮭鱒論 (salmon trout theory) and the politics of non-Western academic terms. The Sociological Review, 68(2), 435–451. 10.1177/0038026120905492

[bibr56-03063127251386080] Kreimer P. (2022a). Constructivist paradoxes part 1: Critical thoughts about provincializing, globalizing, and localizing STS from a non-hegemonic perspective. Engaging Science, Technology, and Society, 8(2), 159–175. 10.17351/ests2022.1109

[bibr57-03063127251386080] Kreimer P. (2022b). Constructivist paradoxes part 2: Latin American STS, between centers and peripheries. Engaging Science, Technology, and Society, 8(3), 87–106.

[bibr58-03063127251386080] Latour B. (2004). Why has critique run out of steam? From matters of fact to matters of concern. Critical Inquiry, 30(2), 225–248.

[bibr59-03063127251386080] Law J. Lin W. (2017). Provincializing STS: Postcoloniality, symmetry, and method. East Asian Science, Technology and Society: An International Journal, 11(2), 211–227. 10.1215/18752160-3823859

[bibr60-03063127251386080] Lewis C. T. Short C. (1907). A new Latin dictionary: Founded on the translation of Freund’s Latin-German Lexicon. American Book Company.

[bibr61-03063127251386080] Liboiron M. Ammendolia J. Winsor K. Zahara A. Bradshaw H. Melvin J. Mather C. Dawe N. Wells E. Liboiron F. Fürst B. Coyle C. Saturno J. Novacefski M. Westscott S. Liboiron G. (2017). Equity in author order: A feminist laboratory’s approach. Catalyst: Feminism, Theory, Technoscience, 3(2), 1–17.

[bibr62-03063127251386080] Lin W. Y. Law J. (2022). Thinking differently with Chinese medicine: ‘Explanations’ and case studies for a postcolonial STS. Social Studies of Science, 52(4), 491–511. 35603800 10.1177/03063127221092180 PMC9315166

[bibr63-03063127251386080] Lynch M. (2017). STS, symmetry and post-truth. Social Studies of Science, 47(4), 593–599. 28791930 10.1177/0306312717720308

[bibr64-03063127251386080] Lyons K. Parreñas J. S. Tamarkin N. Subramaniam B. Green L. Pérez-Bustos T. (2017). Engagements with decolonization and decoloniality in and at the interfaces of STS. Catalyst: Feminism, Theory, Technoscience, 3(1), 1–47. 10.28968/cftt.v3i1.28794

[bibr65-03063127251386080] MacLeod R. (2016). Consensus, civility, and community: The origins of *Minerva* and the vision of Edward Shils. Minerva, 54, 255–292. 10.1007/s11024-016-9305-x

[bibr66-03063127251386080] Mañana-Rodríguez J. (2015). A critical review of SCImago Journal & Country Rank. Research Evaluation, 24(4), 343–354. 10.1093/reseval/rvu008

[bibr67-03063127251386080] Mavhunga C. C. (2017). What do science, technology, and innovation mean from Africa? MIT Press.

[bibr68-03063127251386080] Mason S. Singh L. (2022). When a journal is both at the ‘top’ and the ‘bottom’: The illogicality of conflating citation-based metrics with quality. Scientometrics, 127, 3683–3694. 10.1007/s11192-022-04402-w

[bibr69-03063127251386080] Meloni M. (in press). Racialization and colonial thought in the European Canon: A decolonial perspective. Routledge.

[bibr70-03063127251386080] Mendlowicz M. V. Gekker M. Xavier Gomes de Araújo A. de Oliveira L. Pereira M. G. Berger W. Pires da Luz M. Vilete L. M. Marques-Portella C. Figueira I. Reis da Silva Junior T. (2024). The top-100 cited articles on post-traumatic stress disorder: A historical bibliometric analysis. Psychology, Health & Medicine, 29(3), 453–472. 10.1080/13548506.2022.2147555 36398923

[bibr71-03063127251386080] Mikami K. (2021). The value of the map and the place of STS. Engaging Science, Technology, and Society, 7(2), 76–80. 10.17351/ests2021.827

[bibr72-03063127251386080] Mol A. (2025). Eating is an English world. Duke University Press.

[bibr73-03063127251386080] NatureCulture. (2022, January 3). Other terms, other conditions. NatureCulture (blog). Retrieved October 5, 2025, from https://www.natcult.net/other-terms-other-conditions/

[bibr74-03063127251386080] Neale T. Lancaster K. Addison C. Kearnes M. (2023). What is an STS contribution now? Science, Technology, & Human Values, 48(1), 3–8. 10.1177/01622439221138631

[bibr75-03063127251386080] Neely A. H. Meek L. A. (2024). African experiments in health and healing: Science from the Home and Homestead. Science, Technology, & Human Values, 49(2), 294–317. 10.1177/01622439221119882

[bibr76-03063127251386080] O’Neil D. (2018). English as the lingua franca of international publishing. World Englishes, 37(2), 146–165.

[bibr77-03063127251386080] Open Edition Journals. (n.d.). Revue d’Anthropologie des Connaissances – Informations aux auteurs. Retrieved September 21, 2025, from https://journals.openedition.org/rac/2636#tocto1n7

[bibr78-03063127251386080] Palgrave Macmillan. (n.d.). BioSocieties – For Authors – Submission. Retrieved March 18, 2025, from https://www.palgrave.com/journal/41292/authors/submission

[bibr79-03063127251386080] Park P. K. J. (2013). Africa, Asia, and the history of philosophy: Racism in the formation of the philosophical canon, 1780–1830. State University of New York Press.

[bibr80-03063127251386080] Pranckutė R. (2021). Web of Science (WoS) and Scopus: The titans of bibliographic information in today’s academic world. Publications, 9(1), Article 12.

[bibr81-03063127251386080] Ramírez-Castañeda V. (2020). Disadvantages in preparing and publishing scientific papers caused by the dominance of the English language in science: The case of Colombian researchers in biological sciences. PLOS ONE, 15(9), Article e0238372 10.1371/journal.pone.0238372 PMC7494110 32936821

[bibr82-03063127251386080] Rodriguez Medina L. (2018a). Editorial welcome. Tapuya: Latin American Science, Technology and Society, 1(1), 1–6. 10.1080/25729861.2017.1368626

[bibr83-03063127251386080] Rodriguez Medina L. (2018b). Internationalizing science and technology: Some introductory remarks. Tapuya: Latin American Science, Technology and Society, 1(1), 216–218.

[bibr84-03063127251386080] Rodriguez Medina L. (2024). On epistemic decentralising: Infrastructuring knowledge beyond global North. Globalisation, Societies and Education. Advance online publication. 10.1080/14767724.2024.2307876

[bibr85-03063127251386080] Sage Author Services. (n.d.a). Services we offer. Retrieved September 16, 2025, from https://languageservices.sagepub.com/en/

[bibr86-03063127251386080] Sage Author Services. (n.d.b). English language editing. Retrieved September 16, 2025, from https://languageservices.sagepub.com/en/services/editing.html

[bibr87-03063127251386080] Sage Journals. (n.d.a). Big Data & Society – Submission guidelines. Sage. Retrieved March 18, 2025, from https://journals.sagepub.com/author-instructions/bds

[bibr88-03063127251386080] Sage Journals. (n.d.b). Public Understanding of Science – Submission guidelines. Sage. Retrieved March 18, 2025, from https://journals.sagepub.com/author-instructions/pus

[bibr89-03063127251386080] Sage Journals. (n.d.c). Science, Technology, & Human Values – Submission guidelines. Sage. Retrieved March 18, 2025, from https://journals.sagepub.com/author-instructions/STH

[bibr90-03063127251386080] Sage Journals. (n.d.d). Social Science Information – Journal overview and metrics. Sage. Retrieved March 18, 2025, from https://journals.sagepub.com/overview-metric/SSI

[bibr91-03063127251386080] Sage Journals. (n.d.e). Social Science Information – Submission guidelines. Sage. Retrieved March 18, 2025, from https://journals.sagepub.com/author-instructions/ssi

[bibr92-03063127251386080] Sage Journals. (n.d.f). Social Studies of Science – Submission guidelines. Sage. Retrieved March 18, 2025, from https://journals.sagepub.com/author-instructions/SSS

[bibr93-03063127251386080] Sariola S. (2018). 30th anniversary issue of Science & Technology Studies. Science & Technology Studies, 31(3), 2–4.

[bibr94-03063127251386080] Santos G. D. Sharif N. Xing J. L. (2023). Translating STS in China: Disciplinary struggles and future prospects. Engaging Science, Technology, and Society, 9(1), 23–49.

[bibr95-03063127251386080] Scimago. (2024). SJR—Scimago Journal & Country Rank [Portal]. Retrieved July 7, 2024, from http://www.scimagojr.com

[bibr96-03063127251386080] Schmitz J. R. (2014). Looking under Kachru’s (1982, 1985) three circles model of world Englishes: The hidden reality and current challenges. Revista Brasileira de Linguística Aplicada, 14, 373–411.

[bibr97-03063127251386080] Scopus. (2025). Content policy and selection. Retrieved October 4, 2025, from https://www.elsevier.com/en-au/products/scopus/content/content-policy-and-selection

[bibr98-03063127251386080] Shineha R. Ema A. Tsukahara T. (2010). Work in progress: Proposal for comparative studies on East Asia STS. East Asian Science, Technology and Society: An International Journal, 4(1), 153–160. 10.1215/s12280-010-9123-4

[bibr99-03063127251386080] Singh R. Lynch M. (2025). Proverbial economies of STS. Social Studies of Science, 55(3), 327–349. 10.1177/03063127241294038 39511923

[bibr100-03063127251386080] Sismondo S. (2010). An introduction to science and technology studies (2nd ed.). Wiley-Blackwell.

[bibr101-03063127251386080] Sismondo S. (2017). Post-truth? Social Studies of Science, 47(1), 3–6. 28195024 10.1177/0306312717692076

[bibr102-03063127251386080] SISSA Medialab. (n.d.a). Journal of Science Communication – Editorial Team and Staff. Retrieved September 16, 2025, from https://jcom.sissa.it/site/editorial-team/

[bibr103-03063127251386080] SISSA Medialab. (n.d.b). Journal of Science Communication América Latina – Editorial Team and Staff. Retrieved September 16, 2025, from https://jcomal.sissa.it/site/editorial-team/

[bibr104-03063127251386080] SISSA Medialab. (n.d.c). Journal of Science Communication – Guidelines for Authors. Retrieved September 15, 2025, from https://jcom.sissa.it/site/authors/

[bibr105-03063127251386080] Smith C. Williams E. L. Wadud I. A. Pirtle W. N. , & Cite Black Women Collective. (2021). Cite Black Women: A critical praxis (a statement). Feminist Anthropology, 2(1), 10–17. 10.1002/fea2.12040

[bibr106-03063127251386080] Smith L. A. Torres M. Parrini-Roses R. (2025). Introduction: Knowing borders: Materialidades, ecologías, estéticas. Catalyst: Feminism, Theory, Technoscience, 11(1), 1–23.

[bibr107-03063127251386080] Springer Nature. (n.d.a). Minerva: A review of science, learning and policy – Submission guidelines. Springer. Retrieved March 18, 2025, from https://link.springer.com/journal/11024/submission-guidelines

[bibr108-03063127251386080] Springer Nature. (n.d.b). NanoEthics: Studies of new and emerging technologies - Submission guidelines. Springer. Retrieved March 18, 2025, from https://link.springer.com/journal/11569/submission-guidelines

[bibr109-03063127251386080] Stirling A. (2007). “Opening up” and “closing down”: Power, participation, and pluralism in the social appraisal of technology. Science, Technology, & Human Values, 33(2), 262–294. 10.1177/0162243907311265

[bibr110-03063127251386080] Taylor & Francis Online. (n.d.a). East Asian Science, Technology and Society: An International Journal. Taylor & Francis Group. Retrieved March 18, 2025, from https://www.tandfonline.com/action/authorSubmission?show=instructions&journalCode=teas20

[bibr111-03063127251386080] Taylor & Francis Online. (n.d.b). New Genetics and Society – Instructions for authors. Taylor & Francis Group. Retrieved March 18, 2025, from https://www.tandfonline.com/action/authorSubmission?show=instructions&journalCode=cngs20

[bibr112-03063127251386080] Taylor & Francis Online. (n.d.c). Science as Culture – Instructions for authors. Taylor & Francis Group. Retrieved March 18, 2025, from https://www.tandfonline.com/action/authorSubmission?show=instructions&journalCode=csac20

[bibr113-03063127251386080] Taylor & Francis Online. (n.d.d). Tapuya: Latin American Science, Technology and Society. Taylor & Francis Group. Retrieved March 18, 2025, from https://tapuya.org/for-authors/submission-guidelines/

[bibr114-03063127251386080] Teplitskiy M. Duede E. Menietti M. Lakhani K. R. (2022). How status of research papers affects the way they are read and cited. Research Policy, 51(4), Article 104484. 10.1016/j.respol.2022.104484

[bibr115-03063127251386080] Trompette P. Vinck D. (2009). Retour sur la notion d’objet-frontière [Revisiting the notion of boundary object]. Revue d’anthropologie des connaissances, 31(1), 5–27.

[bibr116-03063127251386080] Turnbull D. (2000). Masons, tricksters, and cartographers comparative studies in the sociology of scientific and indigenous knowledge. Studies in the History of Science, Technology and Medicine. Harwood Academic.

[bibr117-03063127251386080] UFRGS. (n.d.). Sociologias – Information for authors – Guidelines for submission. Retrieved September 21, 2025, from https://seer.ufrgs.br/index.php/sociologias/information/authors

[bibr118-03063127251386080] UNCTAD. (n.d.a). Classifications. United Nations Trade & Development. Retrieved March, 2025, from https://unctadstat.unctad.org/EN/Classifications.html

[bibr119-03063127251386080] UNCTAD. (n.d.b). Total and urban population, annual. United Nations Trade & Development. Retrieved March, 2025, from https://unctadstat.unctad.org/datacentre/reportInfo/US.PopTotal

[bibr120-03063127251386080] UNCTAD. (2022). Handbook of statistics. United Nations Trade & Development. https://unctad.org/publication/handbook-statistics-2022

[bibr121-03063127251386080] Vaccaro G. Sánchez-Núñez P. Witt-Rodríguez P. (2022). Bibliometrics evaluation of scientific journals and country research output of dental research in Latin America using Scimago Journal and Country Rank. Publications, 10(3), Article 26.

[bibr122-03063127251386080] van den Besselaar P. (2000). Communication between Science and Technology Studies journals: A case study in differentiation and integration in scientific fields. Scientometrics, 47(2), 169–193. 10.1023/A:1005686123917

[bibr123-03063127251386080] Verma A. Chitalia V. C. Waikar S. S. Kolachalama V. B. (2021). Machine learning applications in nephrology: A bibliometric analysis comparing kidney studies to other medicine subspecialities. Kidney Medicine, 3(5), 762–767. 34693256 10.1016/j.xkme.2021.04.012 PMC8515072

[bibr124-03063127251386080] Verran H. (2001). Science and an African logic. University of Chicago Press.

[bibr125-03063127251386080] Vucetic S. (2008). The anglosphere: A genealogy of an identity in international relations [Doctoral dissertation, Ohio State University].

[bibr126-03063127251386080] Zwart H. Barbosa Mendes A. Blok V. (2024). Epistemic inclusion: A key challenge for global RRI. Journal of Responsible Innovation, 11(1), Article 2326721.

